# Exploring gut microbiota in adult Atlantic salmon (*Salmo salar* L.): Associations with gut health and dietary prebiotics

**DOI:** 10.1186/s42523-023-00269-1

**Published:** 2023-10-03

**Authors:** Jie Wang, Yanxian Li, Alexander Jaramillo-Torres, Olai Einen, Jan Vidar Jakobsen, Åshild Krogdahl, Trond M. Kortner

**Affiliations:** 1 National Aquafeed Safety Assessment Center, Institute of Feed Research, Chinese Academy of Agricultural Sciences, No.12 Zhongguancun South St, Beijing, China; 2 Faculty of Veterinary Medicine, Norwegian University of Life Sciences, P.O. Box 5003, Ås, 1432 Norway; 3 Cermaq Group AS, Dronning Eufemias gate 16, Oslo, 0191 Norway; 4 Cargill Aqua Nutrition, Prof. Olav Hanssensvei 7A, Stavanger, 4021 Norway

**Keywords:** Atlantic salmon, Gut microbiota, Gut health, *Mycoplasma*, Lactic acid bacteria, Yeast cell wall based-prebiotics

## Abstract

**Background:**

The importance of the gut microbiota for physiological processes in mammals is well established, but the knowledge of their functional roles in fish is still limited. The aims of this study were to investigate associations between variation in taxonomical composition of the gut microbiota and gut health status in Atlantic salmon and to explore possible modulatory effects of dietary prebiotics in one net-pen farm in open water. The fish with initial mean body weight of around 240 g were fed diets based on the same basal composition, either without (Ref diet) or with (Test diet) yeast cell wall based-prebiotics, during the marine production phase from December to September the following year. Sampling was conducted at three sampling time points: January, April, and September, with average water temperature of 3.9 ℃, 3.4 ℃ and 9.6 ℃, respectively.

**Results:**

As the fish progressed towards September, growth, brush border membrane enzyme activities, and the expression in the gut of most of the observed genes involved in immune (e.g., *il8*, *cd4a*, *myd88*, *il1b*, *gilt*, *tgfb*, *cd8b* and *cd3*), barrier (e.g., *zo1*, *occludin*, *ecad*, *claudin25b* and *claudin15*), and metabolism increased significantly. Lipid accumulation in pyloric enterocytes decreased remarkably, suggesting improvement of gut health condition. The growth of the fish did not differ between dietary treatments. Further, dietary prebiotics affected the gut health only marginally regardless of duration of administration. Regarding gut microbiota composition, a decrease in alpha diversity (Observed species, Pielou and Shannon) over time was observed, which was significantly associated with an increase in the relative abundance of genus *Mycoplasma* and decrease in 32 different taxa in genus level including lactic acid bacteria (LAB), such as *Lactobacillus*, *Leuconostoc*, and *Lactococcus*. This indicates that developmental stage of Atlantic salmon is a determinant for microbial composition. Multivariate association analysis revealed that the relative abundance of *Mycoplasma* was positively correlated with gut barrier gene expression, negatively correlated with plasma glucose levels, and that its relative abundance slightly increased by exposure to prebiotics. Furthermore, certain LAB (e.g., *Leuconostoc*), belonging to the core microbiota, showed a negative development with time, and significant associations with plasma nutrients levels (e.g., triglyceride and cholesterol) and gene expression related to gut immune and barrier function.

**Conclusions:**

As Atlantic salmon grew older under large-scale, commercial farm settings, the *Mycoplasma* became more prominent with a concomitant decline in LAB. *Mycoplasma* abundance correlated positively with time and gut barrier genes, while LAB abundance negatively correlated to time. Dietary prebiotics affected gut health status only marginally.

**Supplementary Information:**

The online version contains supplementary material available at 10.1186/s42523-023-00269-1.

## Background

With a yield of 2.7 million tonnes in 2020, Atlantic salmon represented 32.6% of all finfish species raised in marine and coastal aquaculture [1]. In the intensive aquaculture production cycle, Atlantic salmon may sense and respond to a range of biotic and abiotic factors that may alone or together influence fish in general, and in this context intestinal microbial communities in particular [2, 3].

Over the last decade, the field of 16 S rRNA gene sequencing has witnessed significant advancements in user-friendly workflows and cost-effectiveness. Alongside the development of bioinformatics tools, this has led to a profound understanding of the dynamics, taxonomic composition, and functional profiling of the gut microbiota. In mammals, particularly, this knowledge has proven crucial as intestinal bacterial communities have been found to play a significant role in various host physiological processes and disease development. These processes include, but not limited to, nutrient absorption [4], bile acid metabolism [5], immunity [6, 7], lipid metabolism [8], central nervous system [9], as well as being both risk and medical treatment for inflammatory bowel diseases [10, 11].

On the other hand, research on gut microbiota in fish has not progressed as extensively as in mammals. Characterizing gut microbiota and its relations with physiological functions is an important step towards identifying key microbial clades improving gut health [12–14]. However, most fish microbiota studies published so far are descriptive studies on the taxonomic composition and its changes under different experimental conditions including diet, rearing environment, location within the digestive tract, and health status have not been comprehensively investigated [15].

In the specific case of Atlantic salmon, some recent studies have shed light on the associations between gut microbiota and various host responses. These studies have identified that differentially abundant taxa were significantly related to flesh pigmentation [16, 17], lipid metabolism [18, 19], immune responses [20], and gut barrier biomarkers [21]. Such findings highlight the potential importance of the gut microbiota in various aspects of the fish’s health and overall well-being. Moving forward, a significant milestone in fish microbiota research would be the ability to selectively manipulate the microbiota to promote host growth and health.

Given the important roles of gut microbiota, interest of the feed producers has risen to strengthen fish growth and health directly or indirectly via attempts to regulating gut microbiota composition by adding various feed additives, such as prebiotics that are non-digestible fibers and compounds that promote the growth and activity of beneficial microorganismsin the gastrointestinal tract, into the diets [22–24]. Despite several efforts to study feed additives in fish, available scientific literature has important knowledge gaps regarding the effects of administration of prebiotics on fish growth and gut health, as stated in recent reviews [22, 23, 25].

To address some of the pressing knowledge gaps, the aims of this study therefore were twofold. Firstly, we investigated potential associations between the microbiota of the distal intestinal digesta and host gut health status in Atlantic salmon farmed under commercial conditions. Secondly, we explored the effects of applying dietary yeast cell wall based-prebiotics on production performance, gut health, and gut microbiota composition under the same conditions.

## Results

### Growth performance and body indices

**Fig. 1:**
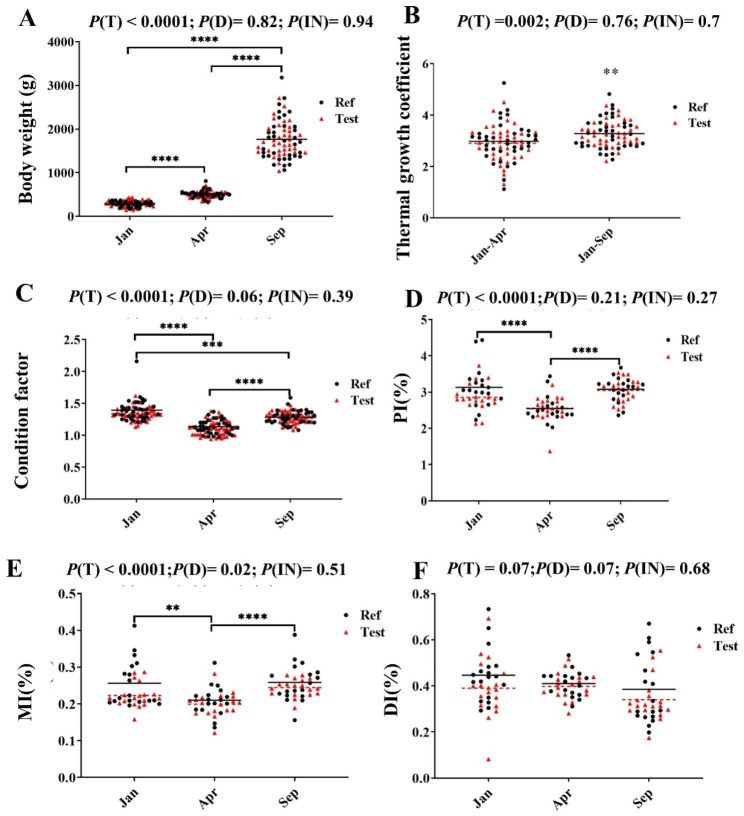
The growth performance, condition factor and organosomatic indices. For thermal growth coefficient, the mean of body weight from Jan-fish was set as initial body weight to calculate Apr-fish and Sep-fish. Black line and red dotted line indicate mean of Ref diet and Test diet in each sampling time points, respectively. Thermal growth coefficient (TGC) = [sampling body weight (g) ^1/3^ - initial body weight (g) ^1/3^] * (∑ day degree)^−1^. Condition factor and organosomatic indices were calculated as: Condition Factor (CF) = 100* body weight (g) / body length^3^ (cm) and Intestinal somatic indices (OSI %) = 100* intestinal tissue weight / body weight (g). *P* < 0.05 (*); *P* < 0.01 (**); *P* < 0.001 (***); *P* < 0.0001 (****)

Regardless of dietary treatments, the fish grew more slowly in the period of January (Jan) to April (Apr), estimated by thermal growth coefficient (TGC), compared to those in the period of Apr to September (Sep) (*P* = 0.002, Fig. 1A and B). No diet effect was found on growth performance (*P* > 0.05, Fig. 1A and B).

Regarding condition factor (CF), the highest values were observed for the Jan-fish, a drop for the Apr-fish, followed by an increase for the Sep-fish (*P* < 0.0001, Fig. 1C). The two treatments showed similar CF values at all sampling time point (*P* > 0.05, Fig. 1C).

For organosomatic indices (OSI) of pyloric intestine (PI), the values decreased from Jan-fish to Apr-fish, then recovering to Jan-fish values for Sep-fish regardless of dietary treatments (*P* < 0.0001, Fig. 1D). The mid intestine (MI) somatic indices showed a similar trend as the PI between sampling time points. Fish fed Test diet had lower MI somatic indices compared to those fed Ref diet (*P* < 0.05, Fig. 1E). Regarding distal intestine (DI), neither sampling time point nor diet significantly affected the somatic index (*P* > 0.05, Fig. 1F).

### Plasma biomarkers

**Fig. 2:**
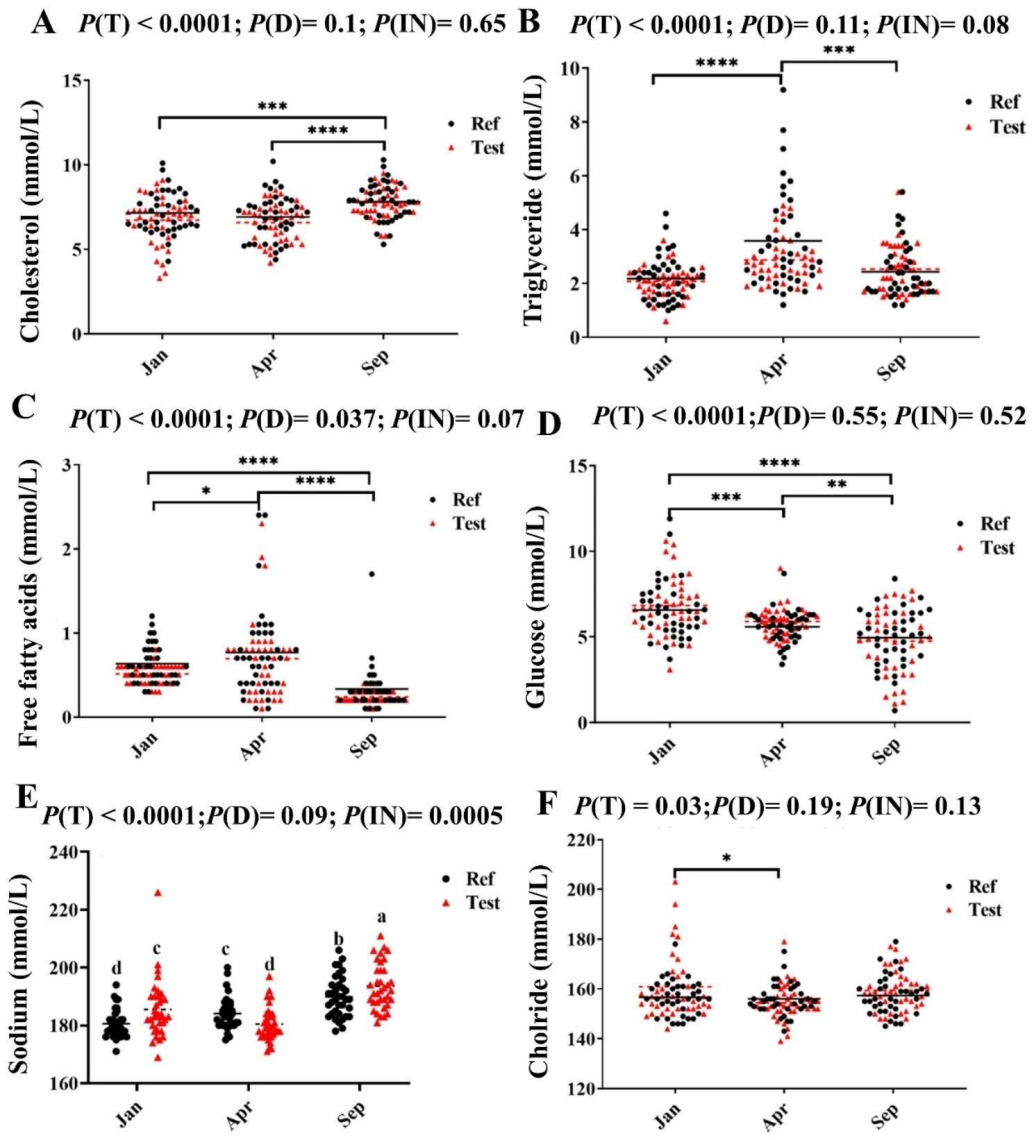
The plasma biochemistry. Black line and red dotted line indicate mean of Ref diet and Test diet in each sampling time points, respectively. For plasma sodium, different letters between values denote significant differences and values sharing the same letters are not significantly different. *P* < 0.05 (*); *P* < 0.01 (**); *P* < 0.001 (***); *P* < 0.0001 (****)

Sep-fish had higher plasma cholesterol level than Jan- and Apr-fish (*P* < 0.0001, Fig. 2A). The plasma triglyceride level increased significantly from Jan to Apr, before decreasing again in Sep-fish to the level of the Jan-fish (*P* < 0.0001, Fig. 2B). Plasma free fatty acids showed similar trend as plasma triglycerides, but the lowest levels were observed for the Sep-fish (*P* < 0.0001, Fig. 2C). The plasma glucose levels showed a decreasing development during the period (*P* < 0.0001, Fig. 2D).

A significant diet effect was observed only for plasma free fatty acids, which was slightly lower in Test compared to the Ref-fed fish (*P* = 0.037, Fig. 2C). However, an interaction effect was observed for plasma sodium level. In Jan the Ref-fed fish showed the lowest level, in Apr the relationship was switched, but the average was similar, whereas in Sep the Test-fed fish again was at a higher level (*P* < 0.0005, Fig. 2E). Regardless of dietary treatment, the plasma chloride levels decreased slightly from Jan- to Apr-fish, before the level increased again in Sep-fish to Jan-fish levels (*P* < 0.05, Fig. 2F).

### Total bile acid and trypsin activities in digesta

Regarding digesta trypsin activities in distal part of proximal intestine (PI 2) and MI, the Jan-fish had higher activities than those of Apr-fish and Sep-fish (*P* < 0.05, Fig S1 A). No clear diet effect was observed at any intestinal segments or sampling time points (*P* > 0.05, Fig S1 A).

Bile acid level in digesta collected in the MI showed a decreasing trend from Jan- to Sep-fish (*P* < 0.05, Fig S1 B), whereas the opposite was the case for proximal part of distal intestine (DI 1) (*P* < 0.05, Fig S1 B). There were no significant diet effects at any intestinal segments or sampling time points (*P* > 0.05, Fig S1 B).

### Specific activity of brush border membrane (BBM) enzyme leucine aminopeptidase (LAP)

**Fig. 3:**
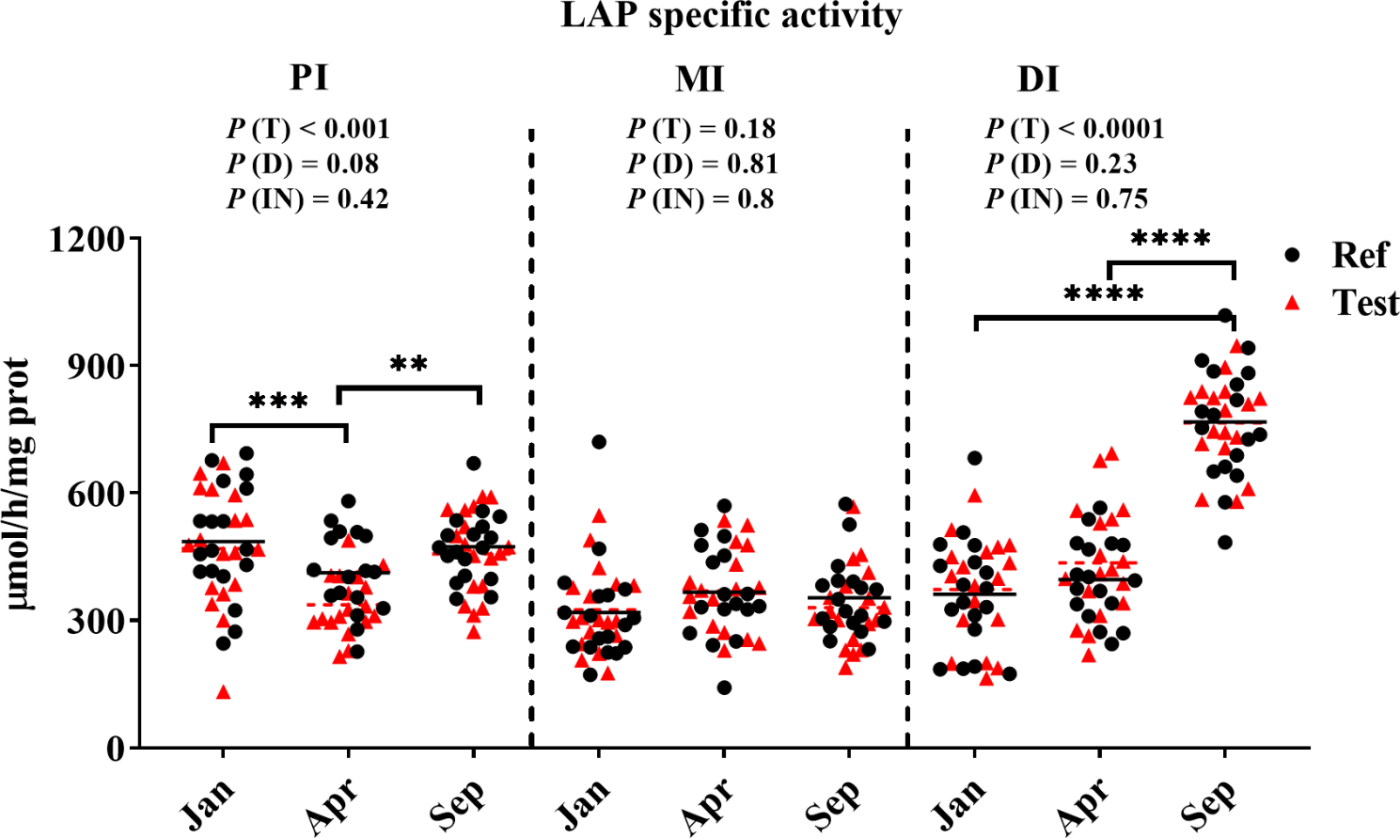
The leucine aminopeptidase (LAP) specific activities in intestinal regions. Black line and red dotted line indicate mean of Ref diet and Test diet in each sampling time points, respectively. *P* < 0.05 (*); *P* < 0.01 (**); *P* < 0.001 (***); *P* < 0.0001 (****)

Specific activity of LAP in the PI showed a decrease from Jan to April before an increase from Apr to Sept (*P* < 0.001, Fig. 3). In the DI, the activity showed similar values in Jan-fish and Apr-fish, followed by a significant increase in Sep-fish (*P* < 0.0001, Fig. 3). In MI, sampling time point did not affect the LAP results significantly (*P* > 0.05, Fig. 3). Diet did not affect the LAP activity at any sampling time points in any of the intestinal regions (*P* > 0.05, Fig. 3).

### Histological characteristics

**Fig. 4:**
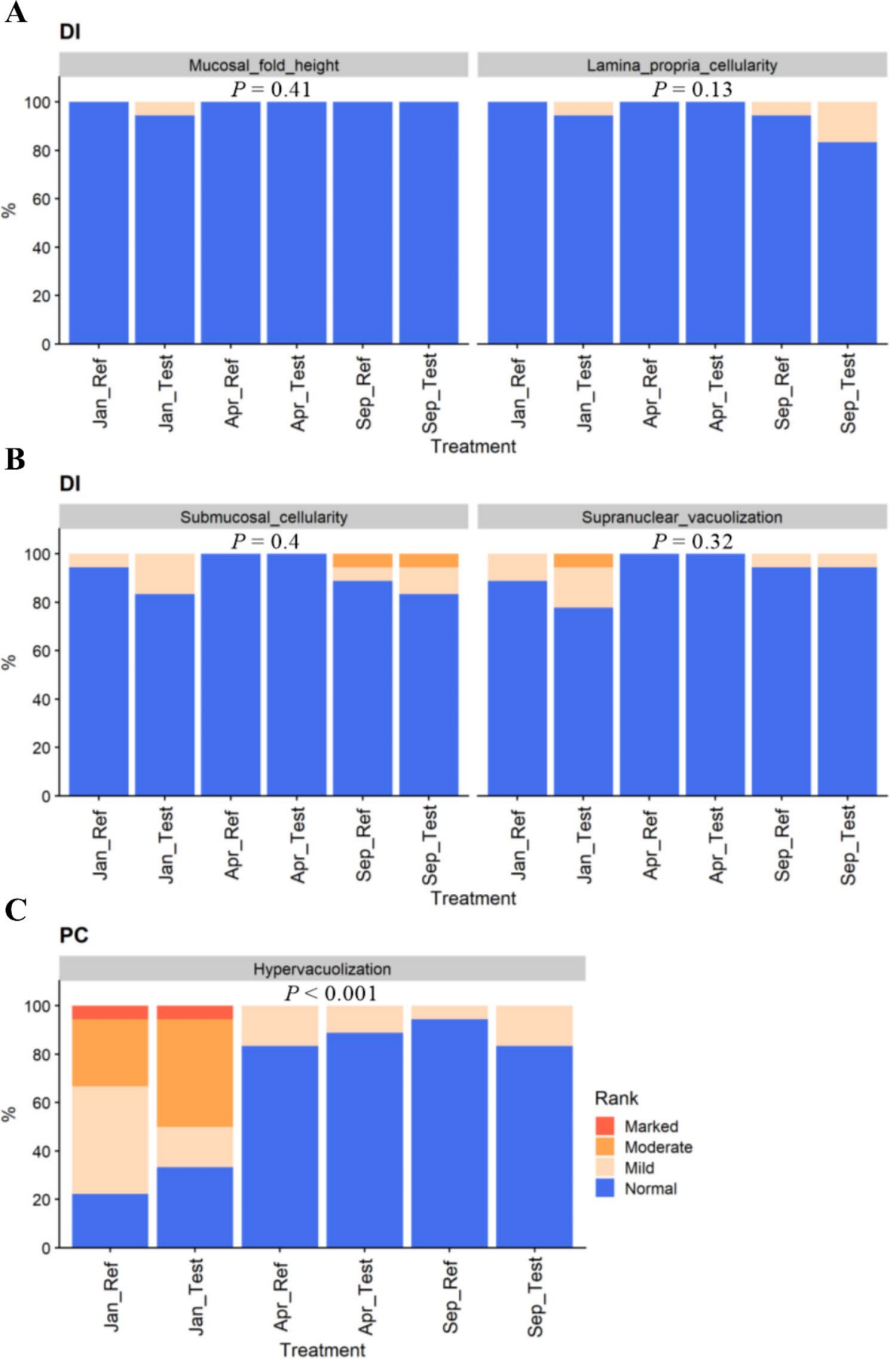
Contingency charts of the distal intestine **(A** and **B)** and pyloric caeca **(C)** morphology results

Regarding the DI, most of the fish showed normal morphological characteristics, and no significant effect of time or diet was observed (*P* > 0.05, Fig. 4A and B). In pyloric caeca (PC), on the other hand, enterocyte hyper-vacuolization, interpreted as steatosis, was observed (Fig. 4C). The symptoms of steatosis were more severe in the Jan-fish (*P* < 0.001, Fig. 4C) compared to those in Apr-fish and Sep-fish which did not show significant difference.

### Gene expression

**Fig. 5:**
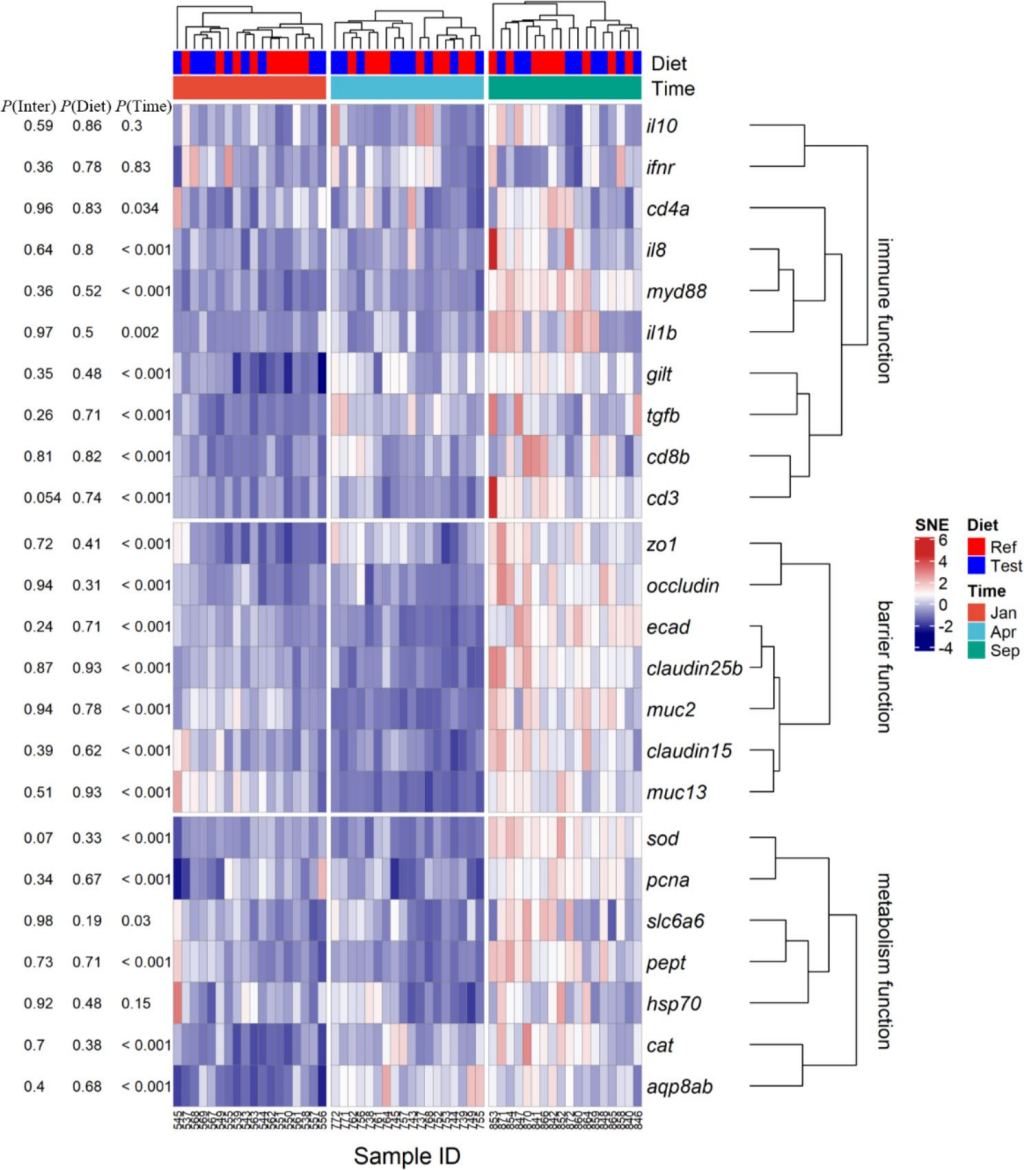
Gene expression profile in the distal intestine. Diet (columns) were clustered based on the Euclidean distance, while gene functions (rows) were clustered based on the Spearman’s rankorder correlation. For cells in the same row, the deeper red color indicates the higher gene expression in each sample; similarly, the deeper blue color indicates the lower gene expression. The annotations for the samples (Diet and Time) are given on the top of the heatmap. The Fig. S2 shows the normalized expression data before scaling. Abbreviations: SNE, scaled normalized expression. The explanations of gene see Table S5

A total of 24 genes related to immune, barrier, and metabolism functions in the DI were profiled (Fig S2). Most of the genes, including immune (*ifnγ*, *il1β*, *tgfβ*, *il10*, *il17a*, *il8*, *cd4α*), barrier (zo*-1*, *claudin-15* and *claudin-25b*) and metabolism functions (*sod*, *cat*, *pcna*, *slc6a6*, *pept* and *aqp8ab*), varied greatly and significantly between sampling time points with the highest expression in the Sep-fish (Fig. 5). No significant diet effects were observed (Fig. 5).

### Gut microbiota

#### Alpha and beta diversity

**Fig. 6:**
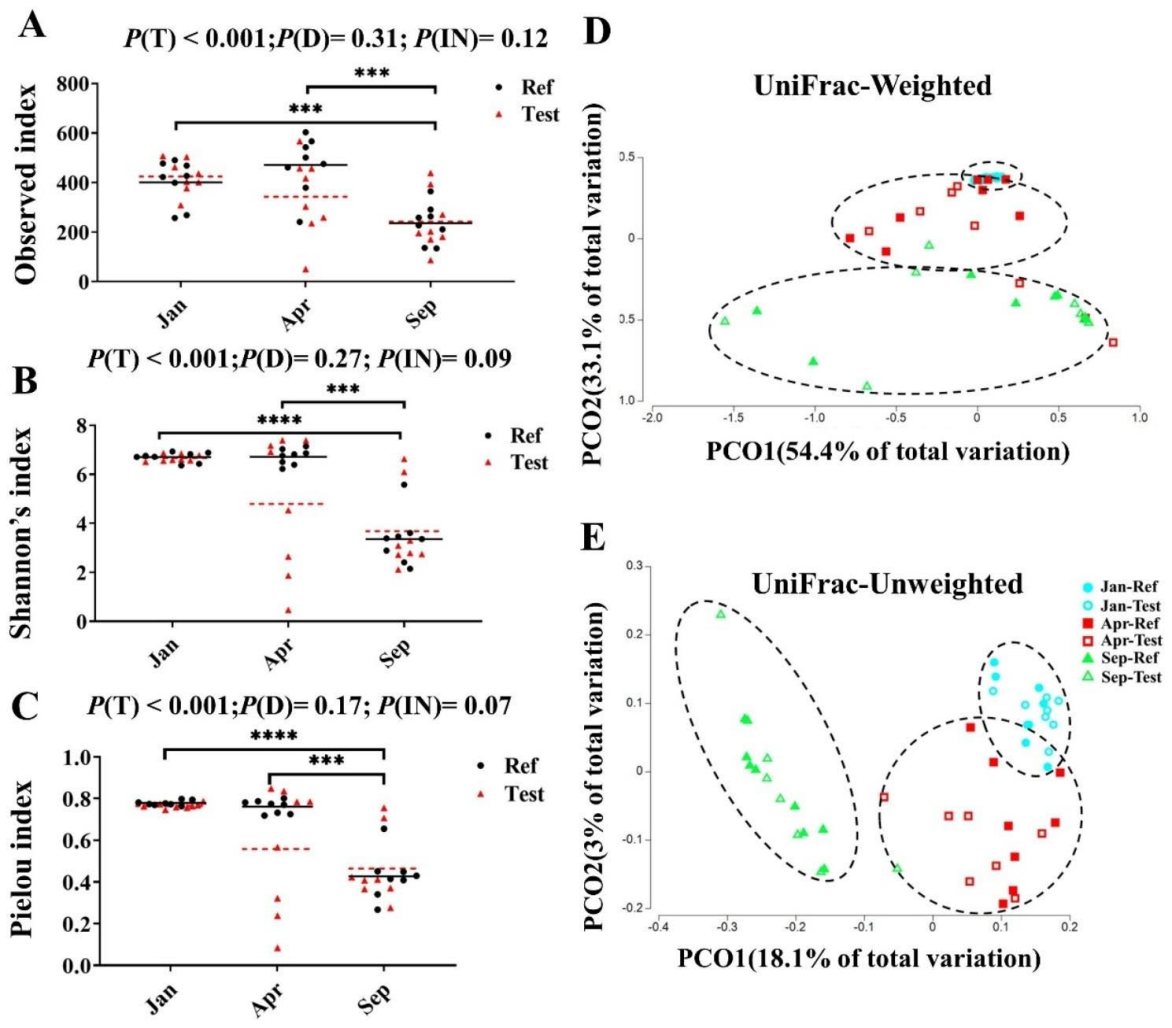
Alpha and beta diversity of distal intestinal digesta microbiome. **(A)** Microbial richness in distal intestinal digesta microbiome of Atlantic salmon, as measured using the Observed species index. **(B)** Microbial diversity in distal intestinal digesta microbiome of Atlantic salmon, as measured using the Shannon’s index. **(C)** Microbial evenness in distal intestinal digesta microbiome of Atlantic salmon, as measured using the Evenness index. **(D)** PCoA plots based on weighted UniFrac show the clustering between treatments. **(E)** PCoA plots based unweighted UniFrac show the clustering between treatments. For beta diversity, each dot represents one sample. Black line and red dotted line indicate mean of Ref diet and Test diet in each sampling time points, respectively. *P* < 0.05 (*); *P* < 0.01 (**); *P* < 0.001 (***); *P* < 0.0001 (****)

The Sep-fish showed the lowest alpha diversity including bacterial richness (Observed species), diversity (Shannon index), and evenness (Pielou index) compared with fish from other sampling time points (*P* < 0.001, Fig. 6A-C). No significant diet effects on alpha diversity were observed (*P* > 0.05, Fig. 6A-C).

Regarding beta-diversity, the results from the pairwise test based on permutation multivariate analysis of variance (PERMANOVA) for both weighted and unweighted UniFrac revealed that the main driver was sampling time point (Table S1). The principal coordinate analysis (PCoA) plots of weighted and unweighted Unifrac showed that the samples within the same sampling time point tended to cluster together and significantly different from other sampling time points. As the fish progressed towards Apr and Sep, individual differences in intestinal community compositions became more marked (Fig. 6D and E).

#### Microbiota composition

**Fig. 7:**
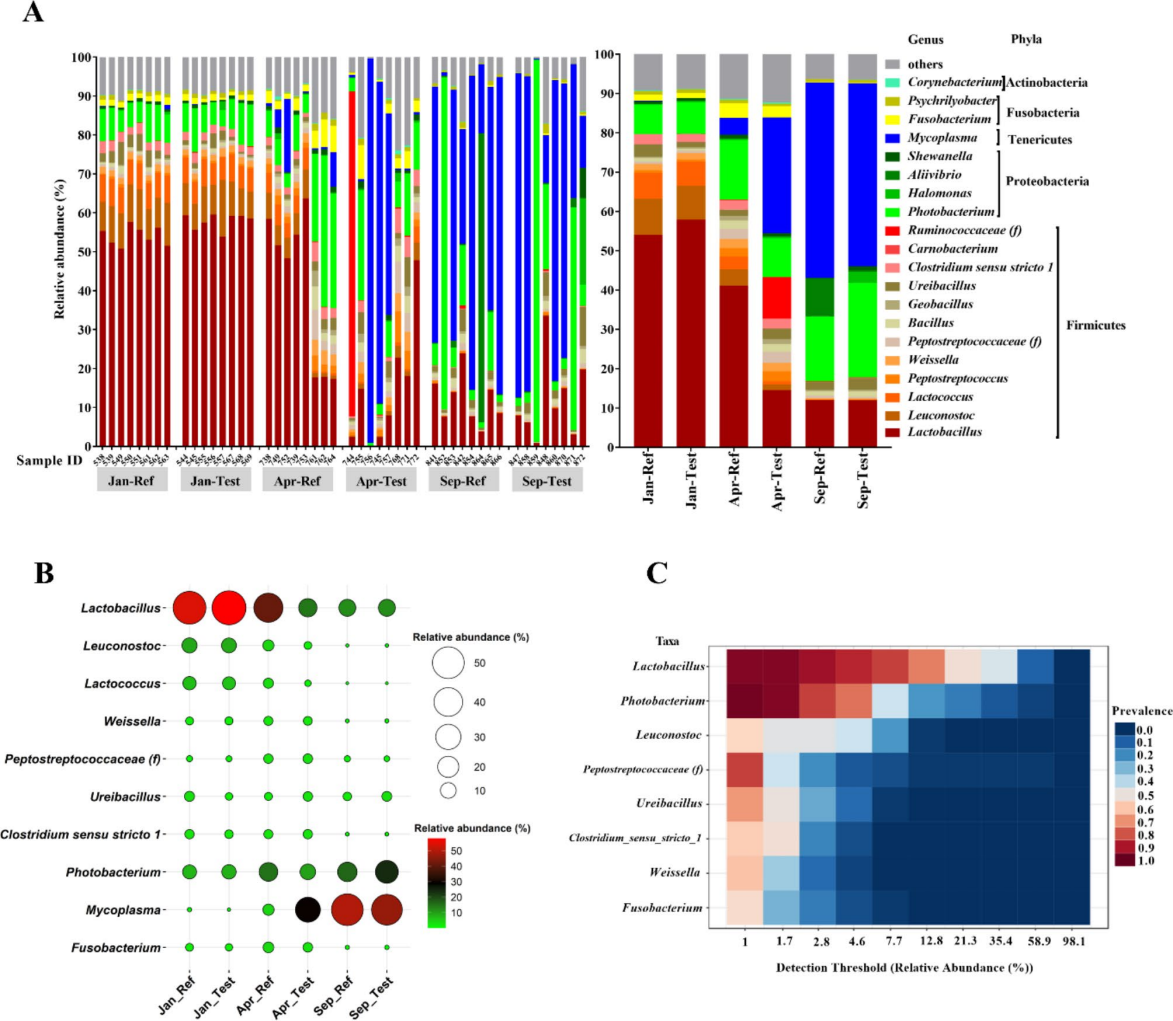
Gut microbiome composition of distal intestinal digesta. **(A)** Top 20 most abundant taxa at genus level of all samples and mean (right side) relative abundance of each taxon. The top 20 genera were selected accounted for more than 80% of the total abundance in each treatment. **(B)** Balloon plot showing the relative abundance of 10 major genera between treatments. The 10 major genera were selected based on MaAsLin 2 and core microbiome analysis. **(C)** The core microbiome between samples at genus level. The figures showing the bacteria were selected above 1% relative abundance in 50% of samples. f, family

A total of 10.8 million counts from the 16 S rRNA gene sequencing were collected with an average of about 75,000 counts per sample. The effective sequences available for downstream analysis after trimming, filtering, and sequence quality screening of ASVs were approximately 16,000 per sample. Twenty-seven phyla were identified in the samples. The relative abundance of all ASVs for the samples are provided in Table S2. Overall, phyla *Firmicutes* (mainly lactic acid bacteria (LAB)), *Proteobacteria* (mainly genus *Photobacterium*), and *Tenericutes* (mainly genus *Mycoplasma*) strongly dominated the gut microbiota and varied between sampling time points (Fig. 7A). As fish progressed towards Apr and Sep, *Mycoplasma* became more prominent, with a corresponding decline in LAB.

More specifically, regardless of diet, Jan-fish were dominated by phylum *Firmicutes*, followed by phylum *Proteobacteria*. The phylum *Firmicutes* was mainly dominated by LAB, with the following results for Ref-Fed fish: *Lactobacillus* (54%), *Leuconostoc* (9%) and *Lactococcus* (7%), and a quite similar picture for the Test-fed fish: *Lactobacillus* (58%), *Leuconostoc* (8%) and *Lactococcus* (6%) dominated. The most abundant genus within phylum *Proteobacteria* was *Photobacterium* showing 8% in both the Ref-fed and Test-fed fish (Fig. 7 and Table S3).

Compared to Jan-fish, Apr-fish showed a slight increase in genera *Proteobacteria* (15% and 10% in Ref-fed and Test-fed fish, respectively) and *Mycoplasma* (4% and 29.5% in Ref-fed and Test-fed fish, respectively), while a decrease in LAB, such as *Lactobacillus* (41%), *Leuconostoc* (4%) and *Lactococcus* (3%) in Ref-fed fish, and *Lactobacillus* (15%), *Leuconostoc* (1%) and *Lactococcus* (1%) in Test-Fed fish, respectively (Fig. 7B and Table S3).

As fish progressed towards Sep, genus *Mycoplasma* (50% and 47% in Ref-fed and Test-fed fish, respectively) became more prominent corresponding to a further reduction in LAB, for example *Lactobacillus* (12%), *Leuconostoc* (0.03%) and *Lactococcus* (0.02%) in Ref-fed fish, and *Lactobacillus* (12%), *Leuconostoc* (0.05%) and *Lactococcus* (0.02%) in Test-Fed fish, respectively (Fig. 7B and Table S3).

#### Core microbiota

Across all samples, 8 genera, i.e., *Lactobacillus*, *Photobacterium*, *Leuconostoc*, *Peptostreptococcaceae (family)*, *Ureibacillus*, *Clostridium_sensu_stricto_1*, *Weissella*, and *Fusobacterium*, were identified as core microbiota based on a threshold above 1% relative abundance and 50% prevalence of all samples. Notably, *Lactobacillus* and *Photobacterium* were present in more than 90% of samples (Fig. 7C).

### Significant associations between microbial clades and sample metadata of interest

**Fig. 8:**
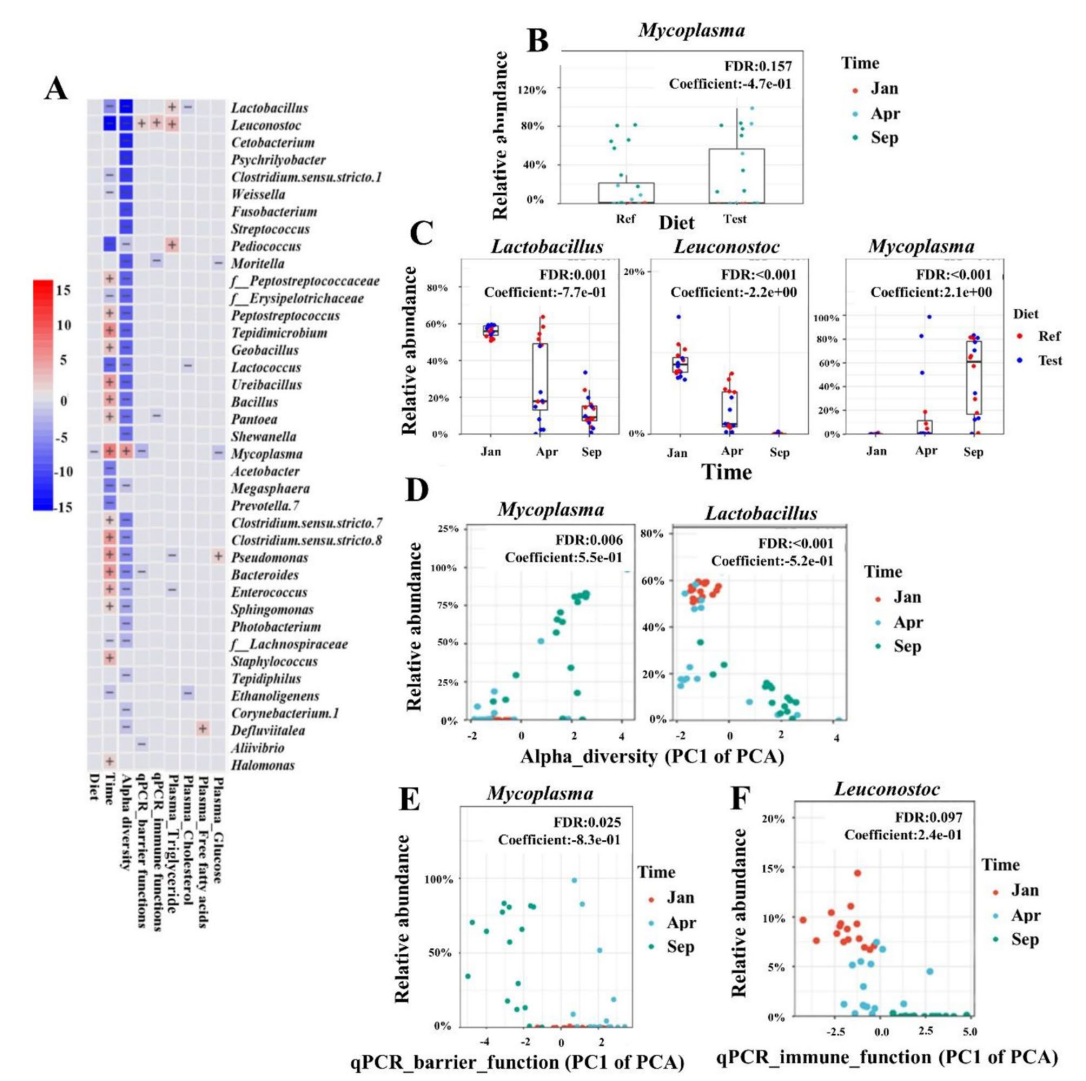
Significant associations between microbial clades with sample metadata. **(A)** Heatmap summarizing all the significant associations between microbial clades and sample metadata. Color key: -log (q-value) * sign (coefficient). Cells that denote significant associations are colored (red or blue) and overlaid with a plus (+) or minus (-) sign that indicates the direction of association. **(B)** Taxa that are more abundant in the Test diet than Ref diet. **(C)** The relative abundance of *Lactobacillus* and *Leuconostoc* showed decreasing trend with time, while the *Mycoplasma* showed increasing trend with time. **(D)** The relative abundance of *Mycoplasma* and *Lactobacillus* were negatively and positively correlated with alpha diversity, respectively. Note that the values of alpha diversity decreased as the PC1 of the PCA increased. **(E)** The relative abundance of *Mycoplasma* showed a clear positive correlation with the gene expressions of gut barrier functions, which decreased as the PC1 of the PCA increased. Note that the expression of the barrier genes decreased as the PC1 of the PCA increased. **(F)** The relative abundance of *Leuconostoc* showed a clear positive correlation with the gene expression of gut immune functions, which increased as the PC1 of the PCA increased. Note that the expression of the immune genes increased as the PC1 of the PCA increased. The significant association was set at FDR (*q-value*) < 0.25. f, family; FDR, false discovery rate

The multivariate association analysis identified 39 differentially abundant taxa with sample metadata of interest (Fig. 8A). The genus *Mycoplasma* was significantly associated with diet, showing slightly higher relative abundances in Test-fed fish than in fish fed Ref diet (FDR = 0.157, Fig. 8B). Twenty-eight taxa showed effect of time (Fig S3), 16 of which showed a positive association with time, including *Mycoplasma* as example in Fig. 8C, while 12 showed a negative association with time, including LAB (e.g., *Lactobacillus* and *Leuconostoc* in Fig. 8C).

There were 33 differentially abundant taxa identified for significant association with alpha-diversity (Fig S4). Notably, the relative abundance of *Mycoplasma* showed a clear negative correlation with the value of alpha diversity, which increased as the PC1 of the PCA increased (FDR = 0.006, Fig. 8D). Another 32 differentially abundant taxa, including LAB (e.g., *Lactobacillus* in Fig. 8D), were positively correlated with alpha diversity, which decreased their abundance as the PC1 of the PCA increased.

Four differentially abundant taxa, including *Mycoplasma*, *Aliivibrio*, *Bacteroides*, and *Leuconostoc*, were found to be associated with the gene expression of gut barrier functions (Fig S5). Notably, the relative abundance of *Mycoplasma* showed a clear positive correlation with the expression of genes involved in gut barrier functions, and increased as the PC1 of the PCA decreased (FDR = 0.025, Fig. 8E).

Three differentially abundant taxa, including *Leuconostoc*, *Moritella*, and *Pantoea*, were found to be associated with the expression of genes involved in immune functions (Fig S6). The relative abundance of *Leuconostoc* showed a clear positive correlation with the expression of these genes, which increased as the PC1 of the PCA increased (FDR = 0.097, Fig. 8F).

The relative abundance of *Lactobacillus*, *Leuconostoc*, and *Pediococcus* were positively correlated, and the relative abundance of *Enterococcus* and *Pseudomonas* were negatively correlated to the plasma triglyceride levels (Fig S7). The relative abundance of *Ethanoligenens*, *Lactobacillus*, and *Lactococcus* was negatively correlated with the plasma cholesterol level (Fig S8). A positive correlation was observed between the relative abundances of *Defluviitalea* and plasma free fatty acid level (Fig S9). Furthermore, the relative abundance of *Mycoplasma* and *Moritella* was negatively correlated with the plasma glucose level, while the relative abundance of *Pseudomonas* was positively correlated with the plasma glucose level (Fig S10).

## Discussion

### General performance

The observed difference in growth between the observation periods, followed the differences in water temperature, as expected [26, 27]. Rate of metabolism in poikilotherm animals follows the temperature and so does growth when feed is offered *ad libitum* [28, 29]. The observed effects on biochemical markers of digestive functions in the intestine as well as for nutrient transport in the blood show effects mainly reflecting the variation in growth [30–32].

The observations of hyper-vacuolization in the enterocytes of the pyloric caeca in the Jan-fish, indicate a steatosis condition, as also observed in our previous study [33]. The prominent symptom of Jan-fish may be related to insufficient supply of choline which recently has been defines as an essential nutrient for Atlantic salmon, and therefore necessary for lipid transport [34, 35]. The choline requirement of Atlantic salmon has not yet been defined for fish kept under conditions as in the present study. Its roles in lipid transport suggest that the requirement is dependent on developmental stage of the fish and water salinity, feed intake, lipid level in the diet, lipid quality, and transport capacity in the fish [35]. Present knowledge suggests that choline level in salmon diets containing high level of plant ingredients often, in the past, has been insufficient to cover the needs under all conditions [34, 35]. The fact that most Apr-fish and Sept-fish showed normal morphological characteristics in the PC indicate that, in these periods in which feed intake was at its lowest and highest, respectively, the choline supply was sufficient for efficient transport of the lipid in the diet.

The results regarding the gene expression in the DI, which for most of them varied greatly between sampling time points with higher expression in the Sep-fish, correspond well with the previous conclusions that these gut functions could be suppressed in winter and elevated in summer [36–39]. The suppression of gut functions in our study could be explained by the complex interaction between harsh external environment (e.g. lower seawater temperature and short daylight) and nutrient availability [40–42]. In summary, in our study most growth and health biomarkers in fish were suppressed in winter, i.e. in January and April, followed by a significant increase thereafter in summer, i.e. in September.

### Gut microbiota and their associations with host gut health responses

Our observation of a significant decrease in alpha diversity in the Sep-fish is in line with the previous studies showing that bacterial richness and diversity tend to decrease in wild as well as farmed Atlantic salmon develop in seawater [19, 43–45]. The decrease in alpha diversity in the Sep-fish reflected decreases in 32 different taxa including LAB (e.g., *Lactobacillus*, *Leuconostoc*, and *Lactococcus*) and a significant increase in *Mycoplasma* abundance. This possibly indicates an adaptive trait during Atlantic salmon developmental progression due to vertically transmitted between generations [46, 47], partly supported by beta diversity showing inter-individual differences in intestinal community composition as the fish grew out. Our results further suggest that relatively low bacterial richness and diversity are not necessarily detrimental for the host, but on the contrary, could be expected in on-growing healthy Atlantic salmon post-smolts and adults [47, 48]. Although this assumption conflicts with previous claims based on observation in human [49], it is strongly supported by improved growth and health biomarkers in Sep-fish, and previous theory that salmon gut can’t filter out bacteria as they age and others dominate [44].

Some relevant scientific literature claim that LAB, as a major component of gut microbiota in Atlantic salmon, is beneficial for the host, at least under certain conditions [50–52]. In the present study, LAB abundance, belonging to the core microbiota, strongly decreased with time, in agreement with earlier observations suggesting that the temperature may be the main driver for this development [53, 54], and the host selection pressure could exert a synergistic effect. It is unknown but likely that the inferior results for gut health biomarkers in Jan-fish and Apr-fish were, at least partly, related to the high population of LAB in our study. This hypothesis seemingly contradicts the beneficial effects of LAB in previous claims, but can be supported by the concerns regarding the efficacy of feeding some *Lactobacillus* strains (reviewed by [55, 56]). For example, *Lactobacillus. plantarum*, which is a potent strain of probiotics, have been found to disrupt the healthy intestinal tissues in humans [57] and worsen colitis in mice [58]. Regarding Atlantic salmon, some studies have also shown that salmon fed soybean meal, replacing fishmeal, inducing soy-induced enteritis in the distal intestine, show high relative abundance of LAB in the digesta of the distal intestine [59, 60]. From these studies, LAB does not appear to protect against immune challenging conditions [61–64]. The apparent discrepancy between our findings and previous claims of beneficial effects of LAB, is that LAB may, directly or indirectly, be involved in physiological functions via intricate functional interconnection between host [55, 56], but the mechanism behind remains unclear.

The genus *Mycoplasma* has been widely reported among the gut microbiota of Atlantic salmon independent of diet composition [65, 66], domestication effect [46, 67], intestinal compartments [68, 69] and environmental conditions [13, 70]. *Mycoplasma* has also been detected sporadically in fish at freshwater stages [44, 71]. In the current study, the *Mycoplasma* tended to increase their relative abundance in the Apr-fish, and thereafter dominated the bacterial communities (about 50%) for the Sep-fish, displaying an important trait of the gut microbiota in post-smolt Atlantic salmon during the development [13, 47]. These findings are in agreement with previous studies that *Mycoplasma* is the most dominant bacteria in post-smolt Atlantic salmon, reaching more than 70% in some case [19, 65, 67, 69]. Despite differences in extrinsic factors (e.g., rearing environment), these extrinsic factors alone cannot explain the differential *Mycoplasma* abundance, as they do not originate from the drinking seawater [72–74] or diet [75]. Certain intrinsic factors, such as physiological status and fish age, are suggested to be the potential reasons for their colonization in intestine, since the *Mycoplasma* seems to keep an important symbiotic relationship with its host [76]. On the other hand, the host was reported to be a determinant for microbial assemblage in Atlantic salmon via filtering specific bacterial communities, including *Mycoplasma* [46, 47].

Several *Mycoplasma* strains can parasitize humans and land-animals, and thereby cause disease [77, 78]. Regarding Atlantic salmon, although the gut microbiota of fish with skin ulcerative disorder was found to be dominated by *Mycoplasma* [45], there is no evidence yet to prove that *Mycoplasma* is responsible for health challenges in fish. On the contrary, like the present study, healthy post-smolt Atlantic salmon typically display high relative abundance of *Mycoplasma* in their gut microbiota, and *Mycoplasma* has therefore been suggested as a potential biomarker for monitoring salmon health [48]. Our study clearly demonstrated that the relative abundance of *Mycoplasma* showed a positive correlation with gut barrier gene expression, possibly suggesting a beneficial effect for the host via increasing intestinal barrier functions. Moreover, the *Mycoplasma* was negatively correlated with plasma glucose levels suggesting a relationship between glucose metabolism and *Mycoplasma* in the salmon. This is in line with one recent study that *Mycoplasma* keeps a synbiotic relationship with the host through some functional signatures, including sugar transporters [76]. Given the indicated important associations of *Mycoplasma* with host physiological functions and its ability to produce arginine, an essential amino acid, which means it is beneficial for disease resistance [79], potential probiotic applications based on *Mycoplasma* strains could be explored for Atlantic salmon in the future.

### Effects of functional additives

The observation in the present study that relative abundance of *Mycoplasma* increased in the Test-fed fish compared to those in fish fed Ref diet may indicate beneficial effects of the prebiotics. However, dietary prebiotics in our study influenced the growth and gut health biomarkers only marginally and independent of the duration of administration. This was unexpected in light of the reports of wide used in aquaculture diets with expectation of enhancement of growth performance, increase digestive enzyme activities, modulation of immune functions and improvement of disease resistance [23, 80]. It is well known that such effects depend on complex interactions between characteristics of the prebiotic themselves, timing and duration of administration, host physiological state, as well as environmental conditions. The explanation for the lack of effects in the present study may therefore be attributed to the fact that knowledge of their mechanism of effects under commercial conditions is limited. Compared to most previous studies conducted in controlled, small-scale experimental trials of limited duration, it is highly likely that, due to more complicated and changeable environmental conditions (e.g., average lower temperature), the effect of prebiotics may not be induced of observed under all commercial conditions [80]. The lack of effect of dietary prebiotics in the present study should be kept in mind and deserves attention to increase the basis for taking decisions regarding how they should be used.

## Conclusions

The main findings of the current study were that most gut health biomarkers and distal intestinal microbial communities varied greatly between sampling time points (alongside season change) with superior physical condition in the Sep-fish. As fish grew older, the genus *Mycoplasma* became more prominent corresponding to a decline in LAB (e.g., *Lactobacillus*, *Leuconostoc*, and *Lactococcus*) causing a decrease in bacterial alpha diversity and an increase in individual differences. Multivariate association analysis showed a significant association between *Mycoplasma* and plasma glucose levels and gut barrier function gene expressions. Certain LAB were significantly associated with gut immune and barrier function gene expressions, as well as with plasma triglyceride and cholesterol levels. Dietary prebiotics influenced the fish only marginally. Our findings fill important knowledge gaps regarding the potential associations between keystone microbes (i.e., *Mycoplasma* and LAB) and host gut health responses in post-smolt Atlantic salmon.

## Materials and methods

### Experimental fish and diets

Atlantic salmon with an initial mean body weight of around 240 g (S.D. = 19) were randomly distributed into 6 commercial sized sea cages (depth: 50 m, perimeter: 200 m), i.e., triplicate cages for per diet (about 55 000 fish per cage). The fish were fed two series of diets (6-mm diameter) based on the same basal composition (47% crude protein, 22% crude lipid, 10% starch, 7% ash and 3% crude fiber, Table S4), either without (Ref diet) or with (Test diet) yeast cell wall based-prebiotics in one to three meals depending on the length of daylight from December 2016 to September 2017in Sommarbukt, near Alta in the far north of Norway. Diets were produced based on the commercial standard procedure of Cargill Aqua Nutrition. The prebiotics were added as the dry meal mix with all other dry ingredients in a homogenous dry mix before the diet mix entered the preconditioner and extruder. The detailed information on the diet composition, such as type and level, are not listed here due to commercial interests and intellectual rights.

### Water parameters

Fish were farmed in open sea cages with naturally seasonal hydrodynamics. A vertically automatic winch (HF5000, Belitronics, Lunde, Sweden) was used to record water temperature, salinity, and oxygen levels at 3 m depth of seawater. The temperature followed natural fluctuations in the seawater, ranging from 2 to 14 °C. Water oxygen and salinity levels ranged from 8 to 15 mg/L, and 12 to 45 ppm, respectively (Fig S11).

### Sampling

**Fig. 9:**
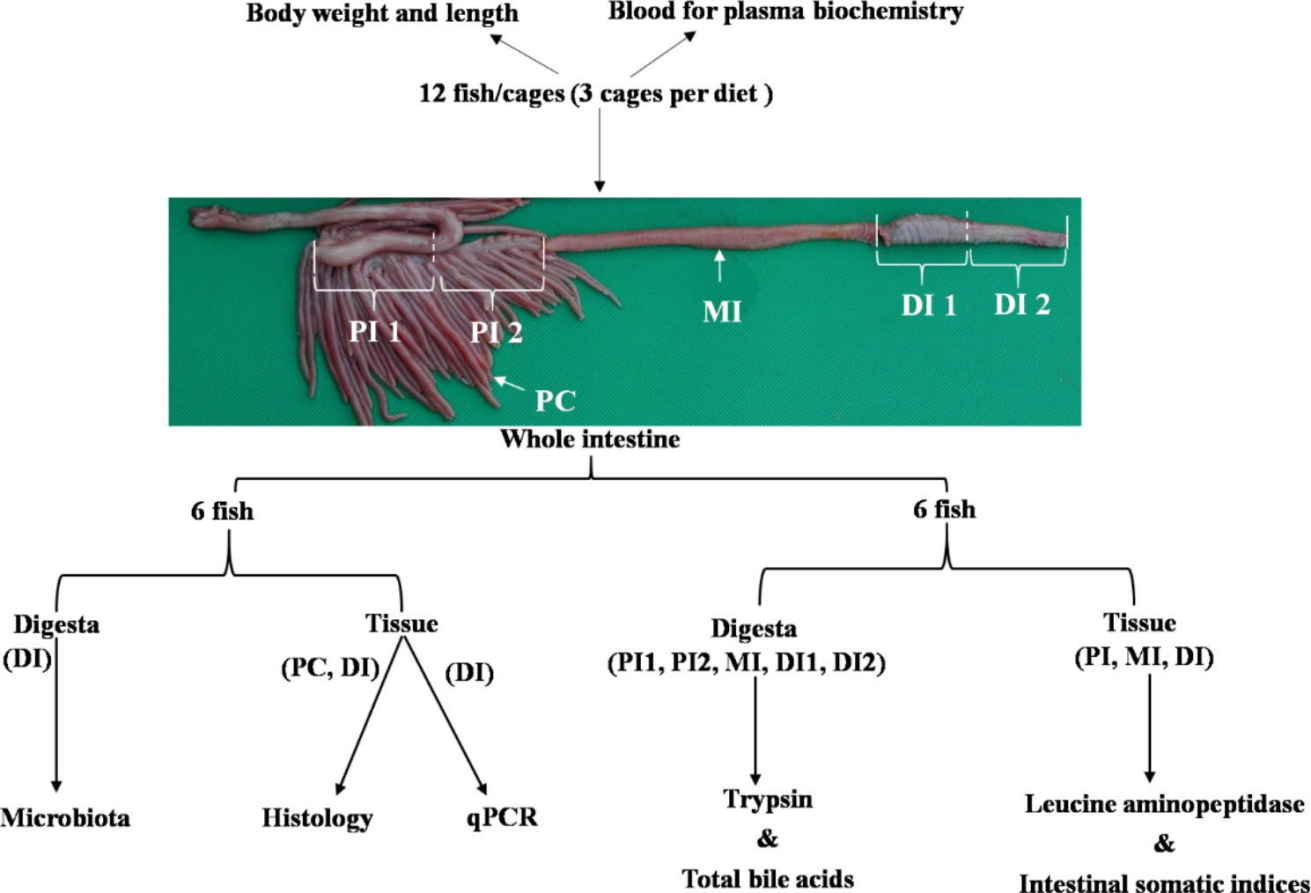
Anatomy of the alimentary tract of Atlantic salmon and workflow of sampling for analysis. Abbreviations: PI: Proximal intestine; PI 1: Proximal part of proximal intestine; PI 2: Distal part of proximal intestine; MI: Mid intestine; DI: Distal intestine; DI 1: Proximal part of distal intestine; DI 2: Distal part of distal intestine; PC: Pyloric caeca

Samples were collected at three time points during production: in Jan, Apr and Sep 2017. At each sampling time point, 12 fish were sampled from each sea cage, i.e., 36 fish per dietary treatment. The fish were euthanized with an overdose of tricaine methane sulfonate before tissue sampling. The anatomy of the alimentary tract of Atlantic salmon and the workflow of sampling are presented in Fig. 9. Growth performance, CF, and plasma biochemistry were measured for all fish (n = 36). The blood was drawn using heparinized vacutainers from the caudal vein. After being spun at 2000 g for 10 min at 4 °C, the plasma was collected, flash-frozen in liquid nitrogen, and then kept at -80 °C pending analysis.

The intestines from 6 to 12 sampled fish per cage, i.e., 18 fish per dietary treatment (n = 18), were removed from the abdominal cavity, cleaned of external fat, and opened longitudinally as illustrated in the bottom-right of Fig. 9. The digesta from intestinal tract was divided in five portions, i.e., the proximal (PI1) and distal (PI2) part of PI, the MI, and the proximal (DI1) and the distal (DI2) part of DI. These five regions of the intestinal tract were divided according to the previous description of Nordrum et al. [81]. The digesta from five sections were collected and pooled for evaluation of bile acid concentration and trypsin activities (n = 3). After removing the digesta, the intestinal tissues of PI, MI, and DI were weighted for intestinal somatic indices (n = 18), respectively, and then used for the analysis of specific activity of the BBM enzyme LAP (n = 18). These samples were frozen in liquid nitrogen and stored at − 80 °C before analysis.

The remaining 6 fish per cage were used for the analysis of histology, qPCR, and microbiota (See the bottom-left of Fig. 9). For histology (n = 18), tissues from DI and PC were collected and fixed in 4% phosphate-buffered formaldehyde solution for one day, and then transferred to 70% ethanol for storage before analysis. Regarding qPCR for gene expression, tissues of DI from 3 fish (n = 9) were taken and preserved in RNAlater solution, incubated at 4℃ for one day, then stored at -20℃ before RNA extraction.

Regarding the microbiota sample collection, methods were carried out as previously reported [75]. Briefly, the DI digesta were scraped and collected into sterile tubes using sterilized tools under a sterile environment created by a gas burner, and then snap-frozen in liquid nitrogen and stored at -80 °C before DNA extraction.

### Plasma biochemistry

Plasma nutrients, including cholesterol, triglyceride, non-esterified (free) fatty acids and glucose, and ions, including chloride and sodium, were analyzed at the Central Laboratory at the Faculty of Veterinary Medicine, Norwegian University of Life Sciences, based on standard procedures (Advia 1800, Siemens Healthcare Diagnostics, Erlangen, Germany).

### Indicators of digestive functions

Trypsin activity assay was conducted as described by Kakade et al. [82] using the substrate benzoylarginine p-nitroanilide (Sigma No. B-4875, Sigma Chemical Co., St. Louis, MO, USA). Bovine trypsin solution was used to make the standard curve. Total bile acid level was analyzed based on the standard procedures from the Enzabile test kit (No. 550,101, BioStat Diagnostic Systems, Cheshire, U.K.) and use of a standard curve based on taurocholic acid solution.

The specific activity of the BBM enzyme LAP was analyzed in intestinal tissue homogenates according to the description of Bieth et al. [83]. The intestinal tissue homogenates were prepared using the ice-cold Tris − mannitol buffer (1:20, w/v). Four-(two-aminoethyl)-benzene-sulfonyl fluoride hydrochloride (Pefabloc SC, Basel, Switzerland) was used as a serine proteinase inhibitor to prevent loss of activity during preparation of the homogenates. Protein concentrations of intestinal tissue homogenates were measured using the BioRad® Protein Assay (BioRad Laboratories, Munich, Germany).

### Histological characteristics

The DI and PC tissue sections, 18 per treatment, were processed according to the standard histological techniques [84] giving 3-µm thickness sections that were stained with hematoxylin and eosin (H&E). The histological characteristics of DI including the length of mucosal fold height, lamina propria cellularity, submucosal cellularity, and supranuclear vacuolization were evaluated by light microscopy and characterized by visual evaluation. The degree of hypervacuolization in PC was evaluated. The histological characteristics were graded using a scoring system with four degrees of change, i.e., normal, mild, moderate and marked, as in our previous study [85].

### Quantitative real-time PCR (qPCR)

At each sampling time point, DI tissues from three fish per cage (n = 9 fish per dietary treatment) were selected for gene expression. DI tissue samples (approximately 100 mg) and homogenised in Trizol reagent (Gibco-Invitrogen Life Technologies) according to the manufacturer’s protocol. Total RNA was extracted using Trizol reagent and PureLink™ RNA Mini Kit according to the the manufacturer’s protocols (Thermo Fisher Scientific). The methods of RNA extraction, RNA purification, DNase treatment, cDNA synthesis, and qPCR assays were conducted according to the MIQE standards [86] and were carried out as previously reported [87]. RNA purity and concentration were measured by Epoch Microplate Spectrophotometer (BioTeK Instruments, Winooski, VT, USA). The 260/280 and 260/230 ratio of all samples were 2.2 (S.D. = 0.03) and 2.3 (S.D. = 0.1), respectively. The RNA integrity was evaluated using the 2100 Bioanalyzer in combination with an RNA Nano Chip (Agilent Technologies, Palo Alto, CA, USA) and 6000 Nano LabChip kit (Agilent Technologies, Palo Alto, CA, USA). The RNA integrity number (RIN value) of all samples was 9.1 (S.D. = 0.9). The RNA polymerase II (*rnapoii*) and hypoxanthine phosphoribosyl transferase 1 (*hprt1*) were evaluated for use as reference genes [88]. The mean normalized levels of target genes were calculated using the plate calibrator-normalised relative raw quantification cycle (Cq) values [89]. The detailed information on primers and genes profiled is shown in Table S5.

### High-throughput sequencing of the gut microbiota

At each sampling time point, 3 fish per cage (the same individuals as those used for qPCR), i.e., 9 fish per dietary treatment and a total of 54 fish, were selected for DNA extraction according to previous suggestions regarding the selection of sample size for microbiota analysis [90].

DNA extraction: One fish from each dietary treatment was randomly selected to divide 54 fish into 9 batches for DNA extraction. About 100 mg of DI digesta from each fish was homogenized using the bead beating following an additional heating step of 95 °C for 7 min as the suggestion by [91]. Hereafter, the homogenized were used for DNA extraction based on the standard procedure provided by the manufacturer of QIAamp Fast DNA Stool Mini Kit (Qiagen, Hilden, Germany). A negative (a DNA extraction blank) and positive control (mock samples, Catalog No: D6300, ZymoBIOMICS Mock Community Standard, Zymo Research) were added to each batch of DNA extraction.

PCR amplification: Since the V1-V2 region showed higher brightness of the bands in the agarose gel than those using V3-V4/V5 regions in our lab [60], the V1-V2 region of the 16 S rRNA gene using 27F (5’ AGA GTTTGA TCM TGG CTC AG 3’) and 338R-I (5’ GCW GCC TCC CGT AGG AGT 3’) and 338R-II (5’ GCW GCCACC CGT AGG TGT 3’) [92] was performed for PCR amplification. The detailed information of PCR was carried out according to our previous descriptions [93]. Briefly, the mixture of 2 µl of DNA template, 22.4 µl PCR Master Mix (Thermo Scientific, CA, USA; catalog no., F531L), 0.3 µl forward (27 F) and 0.3 µl reverse 338R primers (50 pM) was used for PCR. The PCR was run in duplicate with molecular grade water as a negative PCR control. The duplicate PCR products were pooled to evaluate the library preparation by a 1.5% agarose gel electrophoresis. The bright bands of samples between 300 and 350 bp were considered suitable for further analysis. As one of the 9 batches showed the low quality of PCR products, we removed that batch for further analysis (n = 8).

Quantification of 16 S rRNA gene by qPCR: The 16 S rRNA gene quantity in the diluted DNA templates used for the amplicon PCR was measured by qPCR. The qPCR assays were performed using a universal primer set (F, 5’-CCA TGA AGT CGG AAT CGC TAG-3’; R, 5’-GCT TGA CGG GCG GTG T-3’) used for bacterial DNA quantification as previously described [94, 95].

PCR products cleanup, library preparation, and sequencing: PCR product cleanup, library preparation, and sequencing were performed using the standard protocol provided by Illumina (16 S Metagenomic Sequencing Library Preparation) [96]. PCR products were cleaned twice using Agencourt AMPure XP system (Beckman Coulter, Catalog No: A63881) multiplexed by dual indexing using AMPure beads followed the instructions in Nextera XT Index Kit (Illumina, Catalog No: FC-131-1096). Before library normalization, the representative libraries were analyzed using the Agilent DNA 1000 Kit (Agilent Technologies, Catalog No: 5067 − 1505) to verify the library size. Cleaned libraries were quantified using the Invitrogen Qubit™ dsDNA HS Assay Kit (Thermo Fisher Scientific, Catalog No: Q32854), diluted into 4 nM in 10 mM Tris, and pooled in an equal volume. The pooled library was loaded at 6 pM and sequenced with the Miseq Reagent Kit v3 (Illumina, San Diego, CA, USA, Catalog No: MS-102-3003) followed the manufacturer’s instructions.

### Data analysis

#### Statistical analyses of all data with exception of microbiota data

Except for results of trypsin activities, bile acid level, histology, and qPCR, statistical analyses and figures were performed using GraphPad Prism 8 (GraphPad Software, La Jolla, California, United States). Time and dietary treatment were evaluated as class variables in a two-way ANOVA. When interaction effects were significant, one-way ANOVA followed by Tukey multiple comparisons tests were performed to compare the means. Data were evaluated for normality and homogeneity of variance using the normal QQ plot and Shapiro-Wilk test, respectively. When necessary, data were transformed to meet normal distribution.

Regarding trypsin activity and bile acid level, figures were performed using GraphPad Prism 8. Statistical analyses were performed in a two-way ANOVA with time and diet as class variables. Since data did not fulfill the requirement of normal distribution, the Wilcoxon/Kruskal-Wallis test was followed by multiple comparisons tests to compare the means. The values with the same superscript letter are not significantly different. The level of significance was set at *P* < 0.05.

For histology, the scores generated were categorical variables and the differences between the treatments (time_diet) were explored by contingency analysis using the Chisq.post.hoc test. The statistical analyses and figures were performed using the R statistical package (version 4.0.2) within the RStudio interphase (version 1.1.1073; RStudio Inc.).

Regarding the qPCR results, the statistical analyses were performed employing a two-way ANOVA using GraphPad Prism 8. The heatmap figure was made using the *ComplexHeatmap* package [97] within the RStudio interphase.

Individual fish rather than the mean of net pen was used as the statistical unit. Except results of trypsin activity and bile acid level, the level of significance was set at *P* < 0.05 (*); *P* < 0.01 (**); *P* < 0.001 (***); *P* < 0.0001 (****).

#### High-through sequence data processing

Raw sequence reads were demultiplexed, pair-ended, trimmed and denoised using the DADA2 algorithm in QIIME 2 (version 2019.4) to generate amplicon sequence variants (ASVs) [98, 99]. After the sequence denoising, the taxonomy was assigned against the SILVA database (version 132) [100] trained by a naive Bayes machine-learning classifier [101].

#### Quality control

The mock from 8 different DNA extraction batches showed a similar microbiota profile indicating good reproducibility and no significant batch effect (Fig S12). The contaminant sequences were identified based on the two common signatures of contaminants, i.e., frequency inversely relationship with sample DNA levels and the presence in the negative control, as previously described [102]. The removed sequences included the genera *Acinetobacter, Aeromonas*, *Cutibacterium, Flavobacterium*, *Leptothrix, Pseudomonas*, as well as *Chitinophagales* (order), and *Betaproteobacteriales* (order). Moreover, *Streptophyta* was removed, as it is usually assumed as chloroplast sequences [103]. To avoid removal of genuine sequences due to cross-contamination, all removed sequences were double-checked and found most of them were reported in negative controls before [104].

#### Data normalization and analysis

After sequence quality filtering, trimming, filtering of ASVs, the effective sequences were used for further downstream analyses. The alpha diversity was evaluated by the Observed species index (bacterial richness), Pielou index (bacterial evenness), and Shannon’s index (bacterial diversity). The beta diversity between different treatments was performed by Weighted and Unweighted UniFrac distances using the program PRIMER7 (version, 7.0.13) followed by the pairwise test of PERMANOVA to compare each treatment [105]. The core microbiota of all samples was analyzed at genus level (more than 1% relative abundance and 50% prevalence) using the MicrobiomeAnalyst [106].

#### Multivariate associations analysis

The gut microbiota was tested for the associations with sample metadata of interest (Table S6) using the MaAsLin2 (version, 0.99.12) in R with the default parameters [107]. The sample metadata of interest, i.e., alpha diversity (Observed species, Pielou index, and Shannon’s index), plasma cholesterol, plasma triglyceride, plasma free fatty acids, plasma glucose, as well as gene expression related to gut immune and barrier functions, were selected to run the multivariate association testing with two fixed factors (i.e., time and diet). As these three alpha diversity indexes were highly correlated, we ran a principal component analysis (PCA) and extracted the first principal component (PC1) for the association testing to avoid multicollinearity and reduce the number of association testing. The expression of immune and barrier function-related genes were also highly correlated, their extracted PC1 of the PCA was used for the association testing, respectively. Notably, the value of alpha diversity indexes and the levels of gut barrier gene expression were both negatively related to the PC1 values of PCA, which decreased as the PC1 value increased (Table S6). The levels of gut immune gene expression were positively related to the PC1 values of PCA, which increased as the PC1 value increased (Table S6).

### Electronic supplementary material

Below is the link to the electronic supplementary material.

Supplementary Material 1

Supplementary Material 2

Supplementary Material 3

Supplementary Material 4

Supplementary Material 5

Supplementary Material 6

Supplementary Material 7

## Data Availability

The raw sequence data are available in SRA, NCBI: BioProject ID PRJNA662976 (https://www.ncbi.nlm.nih.gov/bioproject/?term=PRJNA662976.). The R scripts for producing our results are made with minor modifications according to Dr. Yanxian Li’s GitHub repository (https://github.com/629yanxianl/Li_AqFl1-Microbiota_2021).

## Abbreviations

LAB: Lactic acid bacteria
16S rRNA: 16 Svedberg ribosomal ribonucleic acid
TGC: Thermal growth coefficient
CF: Condition Factor
OSI: Intestinal somatic indices
PI: Proximal intestine
PI 1: Proximal part of proximal intestine
PI 2: Distal part of proximal intestine
MI: Mid intestine
DI: Distal intestine
DI 1: Proximal part of distal intestine
DI 2: Distal part of distal intestine
PC: Pyloric caeca
BBM: Brush border membrane
LAP: Leucine aminopeptidase
PERMANOVA: Permutation multivariate analysis of variance
PCoA: Principal coordinate analysis
QIIME 2: Quantitative Insights Into Microbial Ecology 2
ASVs: Amplicon sequence variants
ANOVA: Analysis of molecular variance
PCA: Principal component analysis
PC1: The first principal component
