# Supplementary material for: Exploring gut microbiota in adult Atlantic salmon (Salmo salar L.): Associations with gut health and dietary prebiotics
Source: Anim Microbiome. 2023 Oct 3;5:47. doi: 10.1186/s42523-023-00269-1 (PMC10548677; doi:10.1186/s42523-023-00269-1)
Supplement: Supplementary file 1 — Supplementary Material 1 [file 42523_2023_269_MOESM1_ESM.docx]

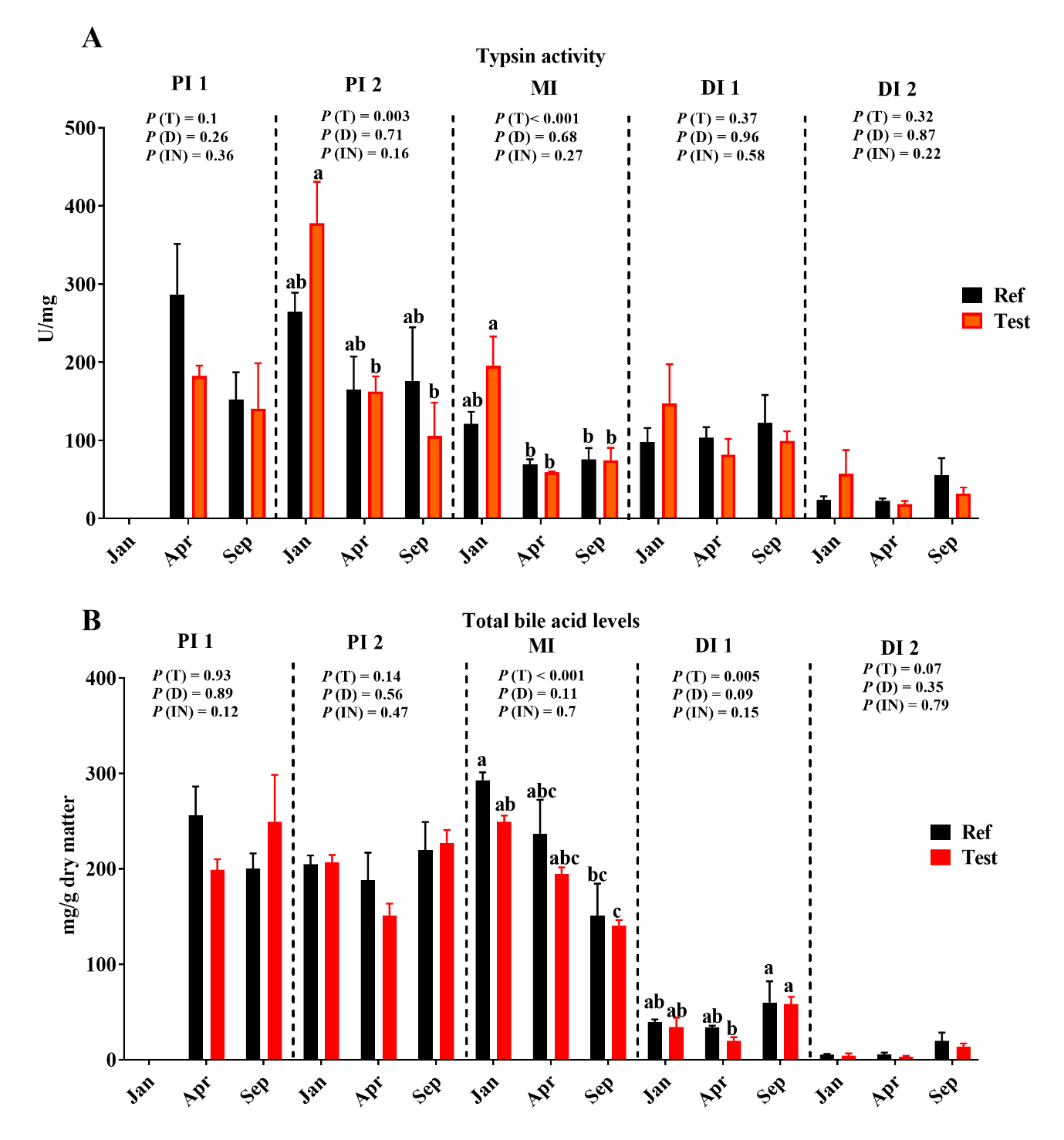
Figure S1. The trypsin activity and total bile acid levels of Atlantic salmon responding to short-term and long-term dietary functional additives. (A) The trypsin activity in the digesta from intestinal sections. (B)The total bile acid levels in the digesta from intestinal sections. Data are presented as mean ± SEM. Different letters between values denote significant differences (*P* < 0.05) and values sharing the same letters are not significantly different.


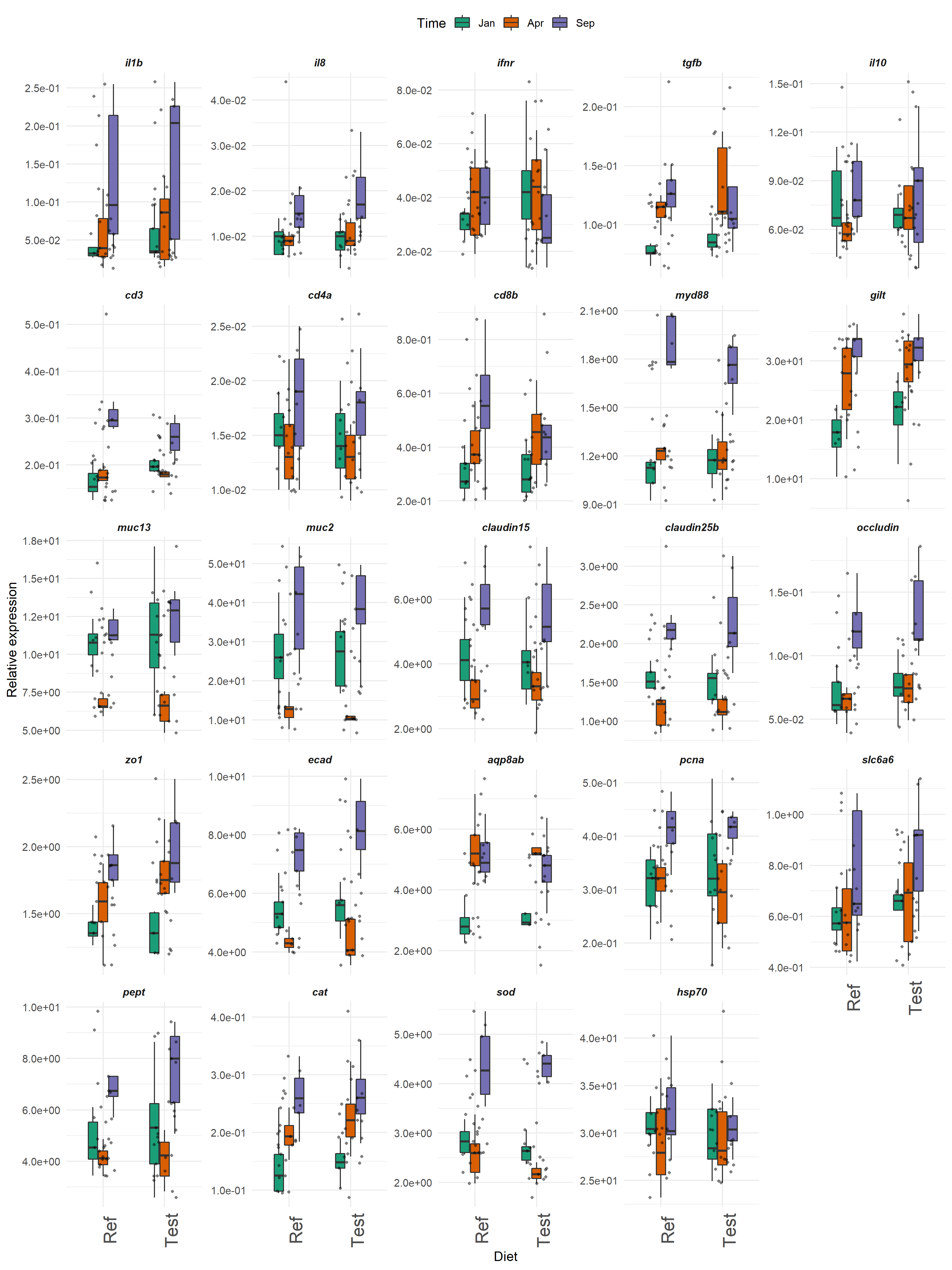


Figure S2: Gene expression profile in the distal intestine of Atlantic salmon responding to short-term and long-term dietary functional additives.

Figure S3: Microbial clades showing significant associations with time. FDR, false discovery rate.


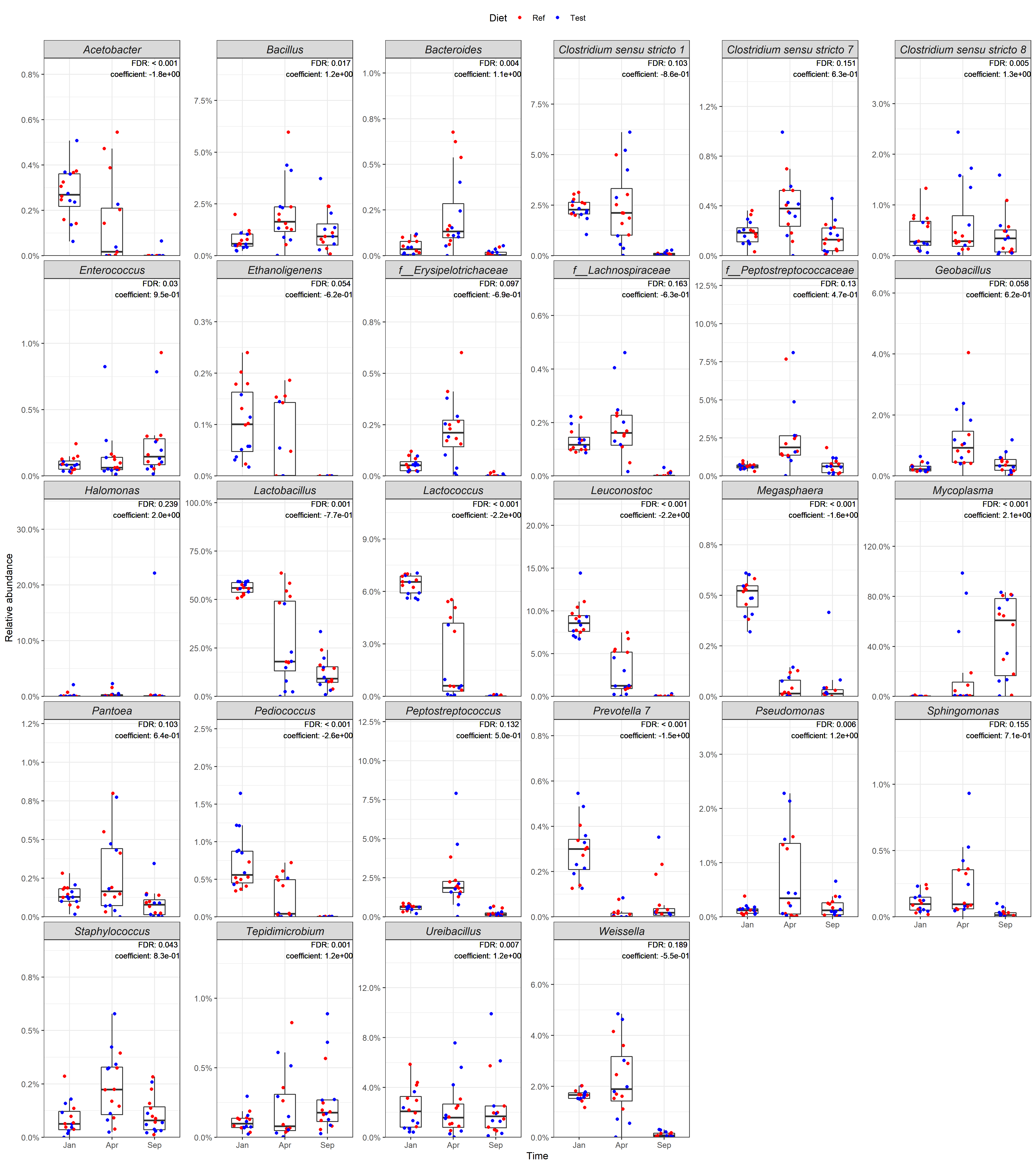


Figure S4: Microbial clades showing significant associations with alpha diversity. Since the alpha diversity were highly correlated, we ran a principle component analysis (PCA) and used the first principle component (PC1) for the association testing to avoid multicollinearity and reduce the number of association testing. The relative abundance of *Mycoplasma* showed a clear negative correlation with alpha diversity, which increased as the PC1 of the PCA increased. Other 32 differentially abundant taxa were positively correlated with alpha diversity, which decreased their abundance as the PC1 of the PCA increased. Note that the values of alpha diversity decreased as the PC1 of the PCA increased. FDR, false discovery rate.


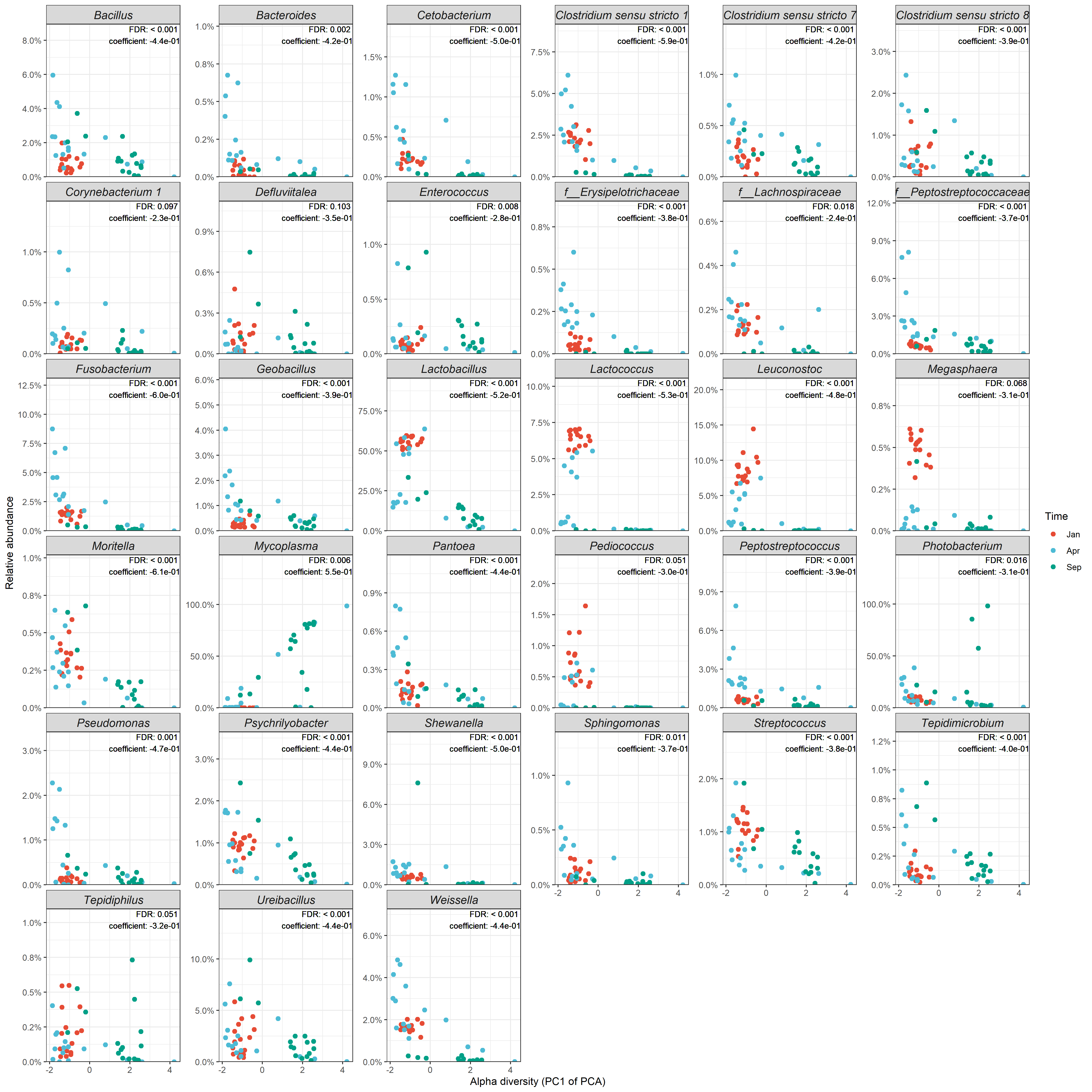

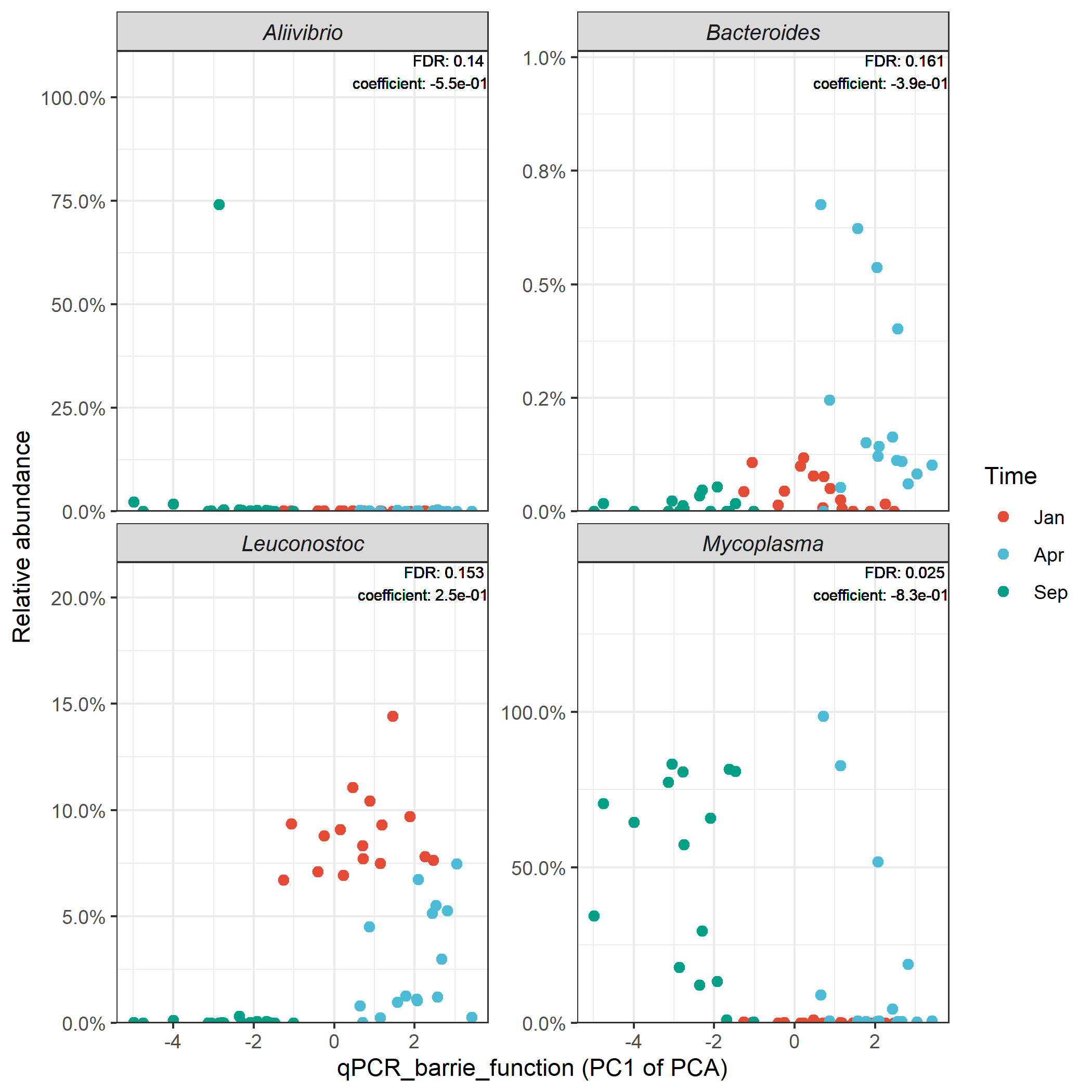


Figure S5: Microbial clades showing significant associations with gut barrier gene expressions. Since the barrier function genes were highly correlated, we ran a principle component analysis (PCA) and used the first principle component (PC1) for the association testing to avoid multicollinearity and reduce the number of association testing. The relative abundance of *Leuconostoc* showed a clear negative correlation with the gene expressions of gut barrier functions, which increased as the PC1 of the PCA increased. Other 3 taxa were positively correlated with gut barrier function gene expressions. Note that the expression levels of the barrier genes decreased as the PC1 of the PCA increased. FDR, false discovery rate.

Figure S6: Microbial clades showing significant associations with gut immune gene expressions. Since the immune function genes were highly correlated, we ran a principle component analysis (PCA) and used the first principle component (PC1) for the association testing to avoid multicollinearity and reduce the number of association testing. The relative abundance of *Leuconostoc* showed a clear positive correlation with the gene expressions of gut immune functions, while other 2 taxa were negatively correlated with the gene expressions of gut immune functions. Note that the expression levels of the immune genes increased as the PC1 of the PCA increased. FDR, false discovery rate.


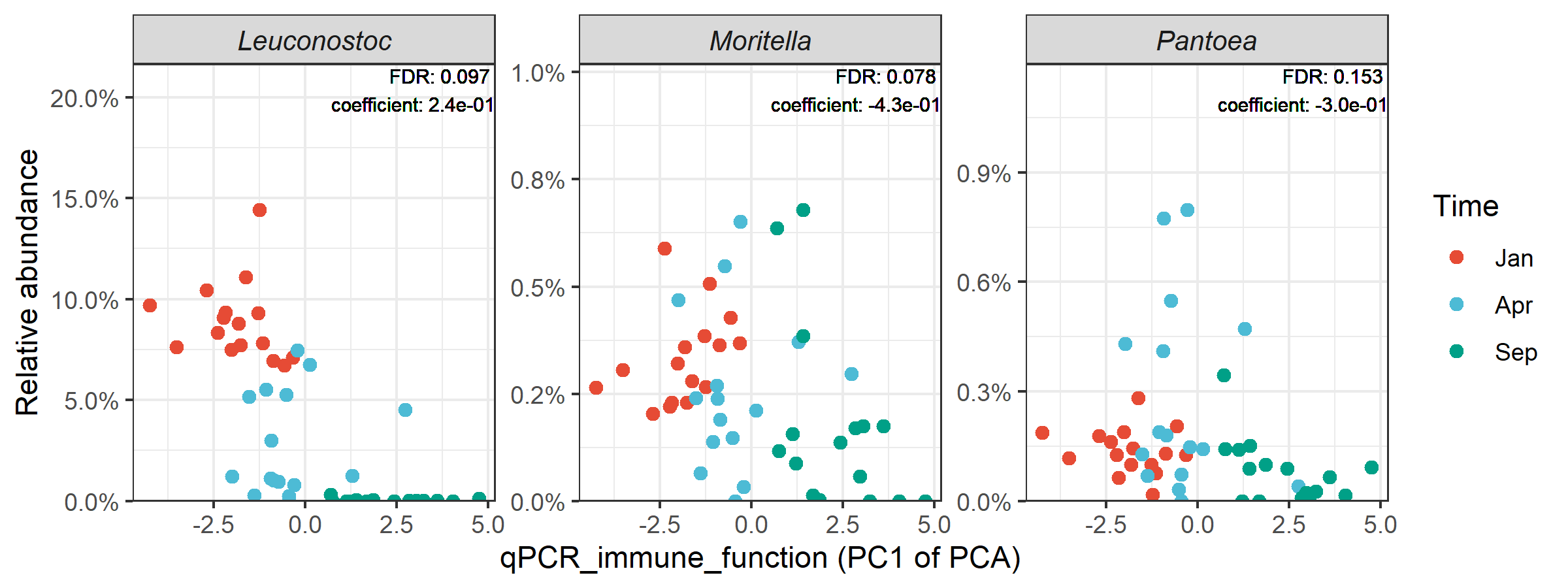

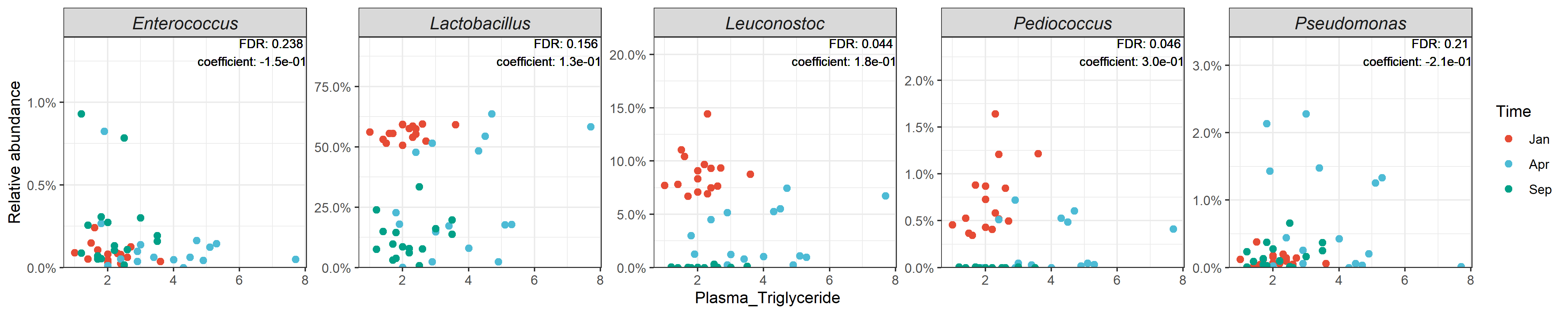


Figure S7. Microbial clades showing significant associations with plasma triglyceride. FDR, false discovery rate.


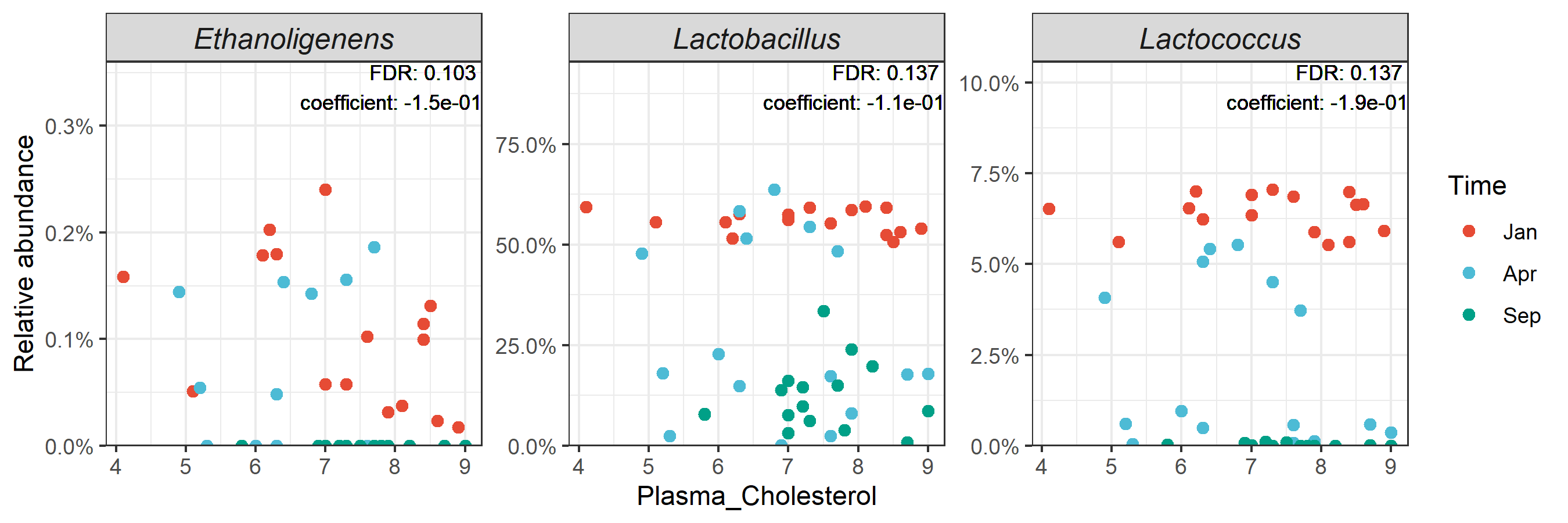


Figure S8. Microbial clades showing significant associations with plasma cholesterol. FDR, false discovery rate.

Figure S9. Microbial clade showing significant associations with plasma free fatty acid. FDR, false discovery rate.


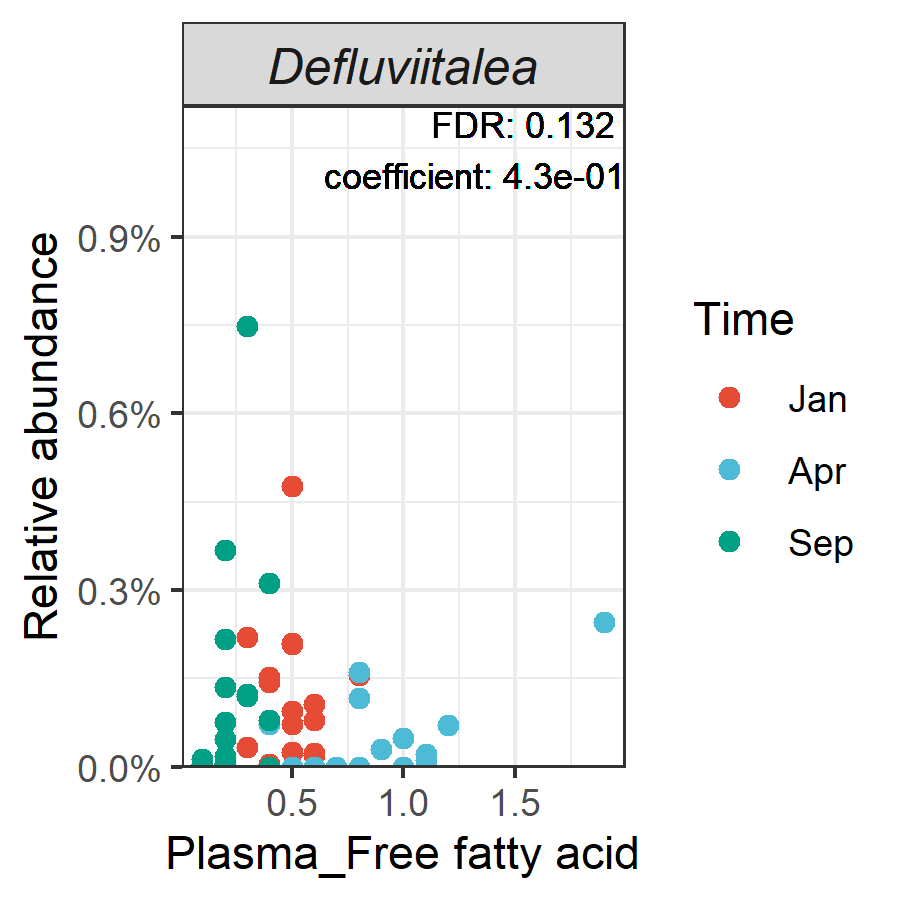

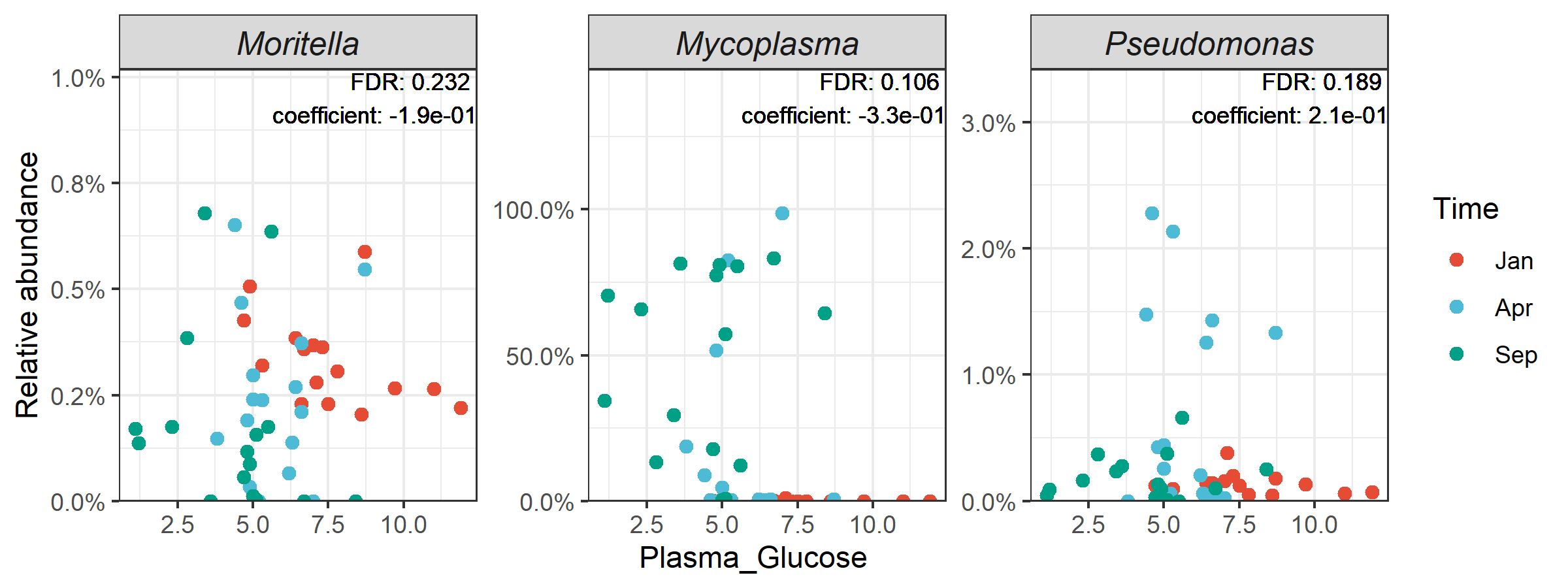


Figure S10. Microbial clades showing significant associations with plasma glucose. FDR, false discovery rate.


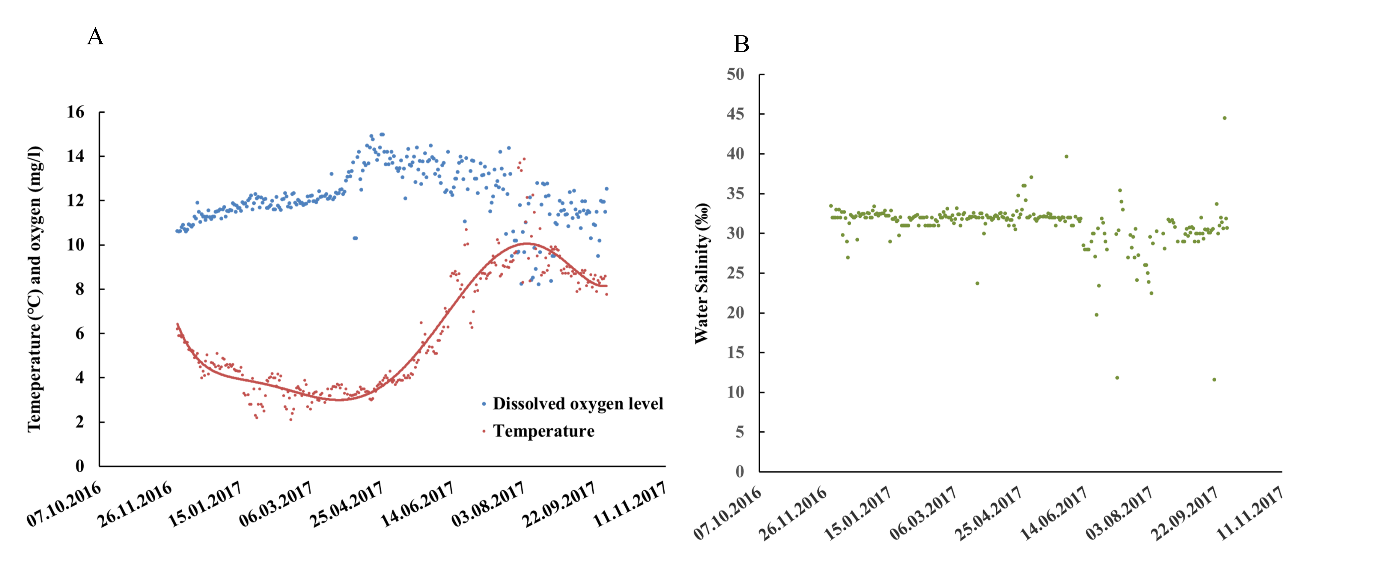
Figure S11: The environmental conditions of water temperature (red dot), oxygen (blue dot) (A), and salinity (green dot) (B) in seawater through experimental period.


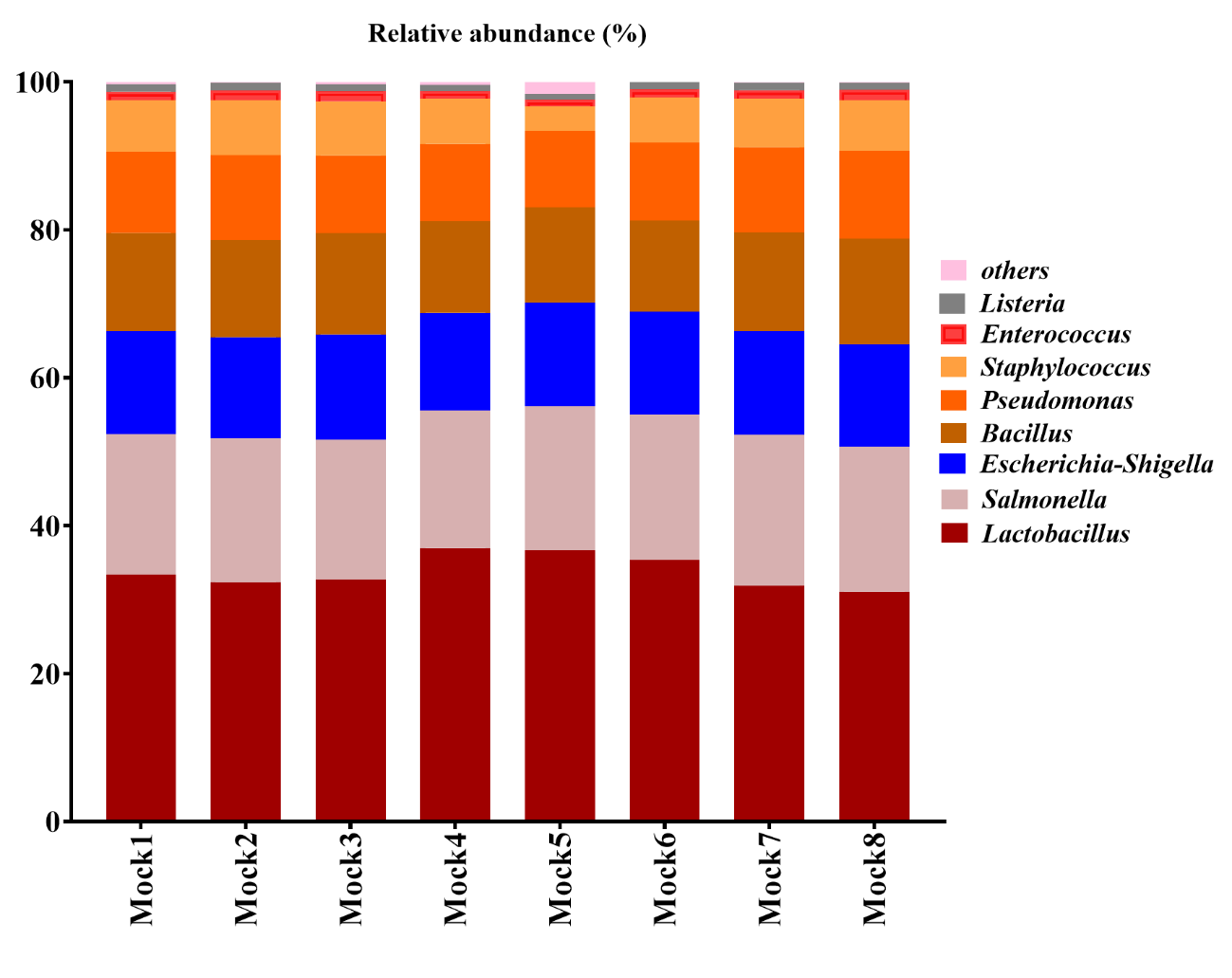
Figure S12: The relative abundance of microbiome in mock samples from 8 DNA extraction batches.
