# Supplementary material for: Exploring gut microbiota in adult Atlantic salmon (Salmo salar L.): Associations with gut health and dietary prebiotics
Source: Anim Microbiome. 2023 Oct 3;5:47. doi: 10.1186/s42523-023-00269-1 (PMC10548677; doi:10.1186/s42523-023-00269-1)
Supplement: Supplementary file 2 — Supplementary Material 2 [file 42523_2023_269_MOESM2_ESM.docx]

| **PERMANOVA** | **Weighted** | | **Unweighted** | |
| --- | --- | --- | --- | --- |
| **Main test** | **Pseudo-F** | ***P* (perm)** | **Pseudo-F** | ***P*(perm)** |
| Diet | 0.45 | 0.70 | 0.97 | 0.42 |
| Time | 8.35 | 0.001 | 6.26 | 0.001 |
| Interaction | 0.65 | 0.68 | 1.00 | 0.39 |
| **Pairwise test** | **t** | ***P* (perm)** | **t** | ***P*(perm)** |
| Jan-Ref vs Jan-Test | 1.41 | 0.108 | 1.04 | 0.177 |
| Jan-Ref vs Apr-Ref | 2.14 | 0.019 | 1.47 | 0.001 |
| Jan-Ref vs Apr-Test | 2.09 | 0.001 | 1.53 | 0.001 |
| Jan-Ref vs Sep-Ref | 2.80 | 0.001 | 2.23 | 0.001 |
| Jan-Ref vs Sep-Test | 2.63 | 0.001 | 2.09 | 0.001 |
| Jan-Test vs Apr-Ref | 2.22 | 0.011 | 1.57 | 0.001 |
| Jan-Test vs Apr-Test | 2.16 | 0.001 | 1.65 | 0.001 |
| Jan-Test vs Sep-Ref | 2.80 | 0.002 | 2.35 | 0.001 |
| Jan-Test vs Sep-Test | 2.63 | 0.001 | 2.21 | 0.002 |
| Apr-Ref vs Apr-Test | 1.28 | 0.193 | 1.08 | 0.178 |
| Apr-Ref vs Sep-Ref | 2.07 | 0.028 | 2.16 | 0.002 |
| Apr-Ref vs Sep-Test | 1.90 | 0.053 | 2.03 | 0.001 |
| Apr-Test vs Sep-Ref | 1.23 | 0.205 | 1.73 | 0.003 |
| Apr-Test vs Sep-Test | 1.18 | 0.222 | 1.61 | 0.001 |
| Sep-Ref vs Sep-Test | 0.13 | 0.975 | 0.87 | 0.976 |

**Table S1.** The PERMANOVA analysis of the Weighted and Unweighted UniFrac between fish fed Ref or Test diet among sampling time point.
