# Supplementary material for: Exploring gut microbiota in adult Atlantic salmon (Salmo salar L.): Associations with gut health and dietary prebiotics
Source: Anim Microbiome. 2023 Oct 3;5:47. doi: 10.1186/s42523-023-00269-1 (PMC10548677; doi:10.1186/s42523-023-00269-1)
Supplement: Supplementary file 4 — Supplementary Material 4 [file 42523_2023_269_MOESM4_ESM.docx]

| Genus Level (%) | Jan-Ref | Jan-Test | Apr-Ref | Apr-Test | Sep-Ref | Sep-Test |
| --- | --- | --- | --- | --- | --- | --- |
| *Lactobacillus* | 54.1 ± 2.5 | 57.9 ± 2.1 | 41 ± 20 | 14.6 ± 15.8 | 12 ± 6.3 | 12 ± 10.6 |
| *Leuconostoc* | 9.1 ± 1.3 | 8.7 ± 2.5 | 4.1 ± 2.7 | 1.4 ± 1.6 | 0.03 ± 0.04 | 0.05 ± 0.1 |
| *Lactococcus* | 6.7 ± 0.3 | 6.13 ± 0.6 | 3.2 ± 2.3 | 0.8 ± 1.4 | 0.02 ± 0.04 | 0.02 ± 0.03 |
| *Weissella* | 1.6 ± 0.3 | 1.7 ± 0.2 | 2.4 ± 1.1 | 2.2 ± 1.8 | 0.09 ± 0.05 | 0.1 ± 0.1 |
| *Peptostreptococcaceae (family)* | 0.7 ± 0.2 | 0.6 ± 0.1 | 2.6 ± 2.1 | 2.8 ± 2.6 | 0.7 ± 0.5 | 0.6 ± 0.4 |
| *Ureibacillus* | 3.2 ± 1.6 | 1.4 ± 1.1 | 1.6 ± 0.9 | 2.7 ± 2.8 | 1.9 ± 1.7 | 2.9 ± 3.4 |
| *Clostridium sensu stricto 1* | 2.6 ± 0.4 | 2.1 ± 0.5 | 2.4 ± 1.2 | 2.5 ± 2.4 | 0.08 ± 0.04 | 0.09 ± 0.1 |
| *Photobacterium* | 7.5 ± 1.7 | 8.1 ± 2.5 | 15.2 ± 13.3 | 10 ± 9 | 16.5 ± 28.4 | 24 ± 35.5 |
| *Mycoplasma* | 0.2 ± 0.3 | 0.04 ± 0.1 | 4.3 ± 6.7 | 29.5 ± 41.9 | 49.8 ± 30 | 46.6 ± 35.1 |
| *Fusobacterium* | 1.6 ± 0.24 | 1.3 ± 0.4 | 3.7 ± 2.2 | 2.8 ± 2.8 | 0.15 ± 12 | 0.18 ± 0.18 |

**Table S3** Relative abundance of major genera between treatments.

Note: Ref: diet without functional ingredients; Test, diet with functional ingredients. The 10 major genera were selected based on MaAsLin 2 analysis and core microbiota. Except *Photobacterium*, they are significant different between life stages. Values expressed in mean ± SD (n=8).
