# Supplementary material for: Exploring gut microbiota in adult Atlantic salmon (Salmo salar L.): Associations with gut health and dietary prebiotics
Source: Anim Microbiome. 2023 Oct 3;5:47. doi: 10.1186/s42523-023-00269-1 (PMC10548677; doi:10.1186/s42523-023-00269-1)
Supplement: Supplementary file 5 — Supplementary Material 5 [file 42523_2023_269_MOESM5_ESM.docx]

**Table S4** Formulation and nutrient composition of two series of diet.

| **Feed composition** | **Ref** | **Test** |
| --- | --- | --- |
| **Ingredients (%)** |  | |
| Marine protein sources**^1^** | 33 | 33 |
| Vegetable protein sources**^2^** | 40 | 40 |
| Fish oil | 9 | 9 |
| Vegetable oil | 8 | 8 |
| Binders & Micro-nutrients | 10 | 10 |
| Sum | 100 | 100 |
| **Nutrient composition (%)** |  | |
| Crude protein | 47 | 47 |
| Crude fat | 22 | 22 |
| Starch | 10 | 10 |
| Crude fiber | 3 | 3 |
| Ash | 7 | 7 |
| **Functional ingredients^3^** |  | |
| Prebiotics |  | √ |

**^1^**Mix of Scandinavian origin fish meal and, fish protein concentrate (Norway). **^2^**Mix of soy protein concentrate, wheat protein concentrate, wheat gluten, sunflower meal. **^3^**Inclusion levels were determined according to recommendations from the producers and not listed here for commercial interests and production of intellectual rights.
